# Sequence-Function Relationships in Phage-Encoded Bacterial Cell Wall Lytic Enzymes and Their Implications for Phage-Derived Product Design

**DOI:** 10.1128/JVI.00321-21

**Published:** 2021-06-24

**Authors:** Roberto Vázquez, Ernesto García, Pedro García

**Affiliations:** a Departamento de Biotecnología Microbiana y de Plantas, Centro de Investigaciones Biológicas Margarita Salas, Madrid, Spain; b Centro de Investigación Biomédica en Red de Enfermedades Respiratorias, Madrid, Spain; University of Kentucky College of Medicine

**Keywords:** endolysins, bacteriophages, bacteriophage therapy, genomics, bioinformatics, antimicrobial agents

## Abstract

Phage (endo)lysins are thought to be a viable alternative to usual antibiotic chemotherapy to fight resistant bacterial infections. However, a comprehensive view of lysins’ structure and properties regarding their function, with an applied focus, is somewhat lacking. Current literature suggests that specific features typical of lysins from phages infecting Gram-negative bacteria (G−) (higher net charge and amphipathic helices) are responsible for improved interaction with the G− envelope. Such antimicrobial peptide (AMP)-like elements are also of interest for antimicrobial molecule design. Thus, this study aims to provide an updated view on the primary structural landscape of phage lysins to clarify the evolutionary importance of several sequence-predicted properties, particularly for the interaction with the G− surface. A database of 2,182 lysin sequences was compiled, containing relevant information such as domain architectures, data on the phages’ host bacteria, and sequence-predicted physicochemical properties. Based on such classifiers, an investigation of the differential appearance of certain features was conducted. This analysis revealed different lysin architectural variants that are preferably found in phages infecting certain bacterial hosts. In particular, some physicochemical properties (higher net charge, hydrophobicity, hydrophobic moment, and aliphatic index) were associated with G− phage lysins, appearing specifically at their C-terminal end. Information on the remarkable genetic specialization of lysins regarding the features of the bacterial hosts is provided, specifically supporting the nowadays-common hypothesis that lysins from G− usually contain AMP-like regions.

**IMPORTANCE** Phage-encoded lytic enzymes, also called lysins, are one of the most promising alternatives to common antibiotics. The potential of lysins as novel antimicrobials to tackle antibiotic-resistant bacteria not only arises from features such as a lower chance to provoke resistance but also from their versatility as synthetic biology parts. Functional modules derived from lysins are currently being used for the design of novel antimicrobials with desired properties. This study provides a view of the lysin diversity landscape by examining a set of phage lysin genes. We have uncovered the fundamental differences between the lysins from phages that infect bacteria with different superficial architectures and, thus, the reach of their specialization regarding cell wall structures. These results provide clarity and evidence to sustain some of the common hypotheses in current literature, as well as making available an updated and characterized database of lysins sequences for further developments.

## INTRODUCTION

Since the antibiotic pipeline started drying up, a worrying increase in the antibiotic-resistant fraction of bacterial populations has been reported (1, 2), and high percentages of antibiotic-resistant populations have been sustained (3, 4). If this situation continues, the cost, both economic and in human lives, will be enormous due to the lack of effective treatments (5, 6). This has prompted the interest in novel antimicrobial development by many public health actors, such as international oversight organizations (7), public health and disease control agencies (3, 4), governments (8), researchers, and several companies (9). Some of the current efforts to gather a new antimicrobial armamentarium have led science toward bacteriophages (10, 11).

To allow the dissemination of their progeny, double-stranded DNA phages provoke lysis of the bacterial host, which is fundamentally accomplished by degradation of the peptidoglycan. This polymer is an essential constituent of the bacterial cell wall, and the breakage of specific bonds within its three-dimensional mesh leads to bacterial death, largely by osmotic shock. The main phage molecule responsible for peptidoglycan degradation is the lysin (also referred to as endolysin). Lysins are released toward their polymeric target, usually with the assistance of another kind of protein, the holin, which create pores in the plasma membrane and thus allow lysin leakage to the periplasm (12). Other phage products collaborate in hampering the cell wall in some of its particular settings; for example, lysins B detach the arabino-mycolyl outer layer of mycobacteria and their relatives (e.g., *Rhodococcus* and *Corynebacterium*) (13). In addition, in some Gram-negative bacteria (G−), effective lysis also needs the concurrent participation of additional phage products named spanins (14). This reveals the significant amount of genetic resources allocated by phages to overcome the barriers that the bacterial cell walls represent.

**FIG 1:**
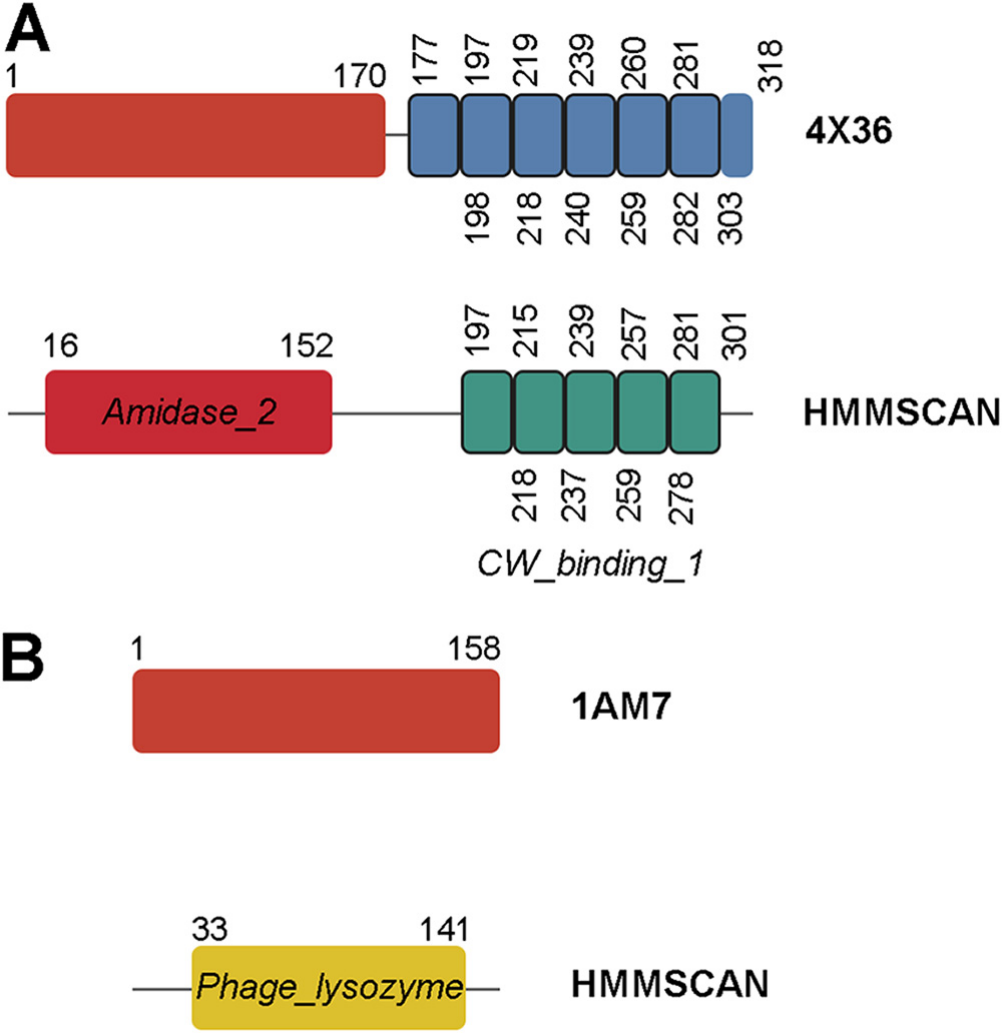
Schematic examples of the architecture of peptidoglycan hydrolases. The top row in each panel corresponds to the domain structure based on the three-dimensional folding according to the corresponding PDB entry (red boxes are EADs; blue boxes are CWBD repeats). In the bottom rows, the domain structure predicted using HMMSCAN is shown. Numbers indicate the amino acid coordinates. (A) LytA autolysin from S. pneumoniae. Note that only five choline-binding repeats (*CW_binding_1*) of the six present in the three-dimensional structure are predicted. (B) Lysozyme from lambda coliphage.

In addition to using whole phage particles as therapeutic agents against bacterial infections (so-called phage therapy), current efforts also point out to artificially repurposing certain phage products, such as lysins, as antimicrobials (enzybiotics) (11, 15, 16). The concept is rather simple: the external addition of purified lysins to a susceptible bacterium would cause bacterial lysis whenever the lysin degrades the peptidoglycan. This process is straightforward in the case of Gram-positive bacteria (G+), and the therapeutic effect of enzybiotics on G+ has been fully confirmed experimentally (15). The most important characteristics that make enzybiotics amenable to be developed as therapeutics are (i) a certain specificity for the original bacterial host and some closely related bacteria, which would prevent normal microbiota from being harmed (16, 17), or, conversely, the possibility of broad-range lysins, if needed (18); (ii) a lower chance of provoking the appearance of resistant bacteria, which is speculated to be due to the essential nature of the highly conserved peptidoglycan (that is, changes in its structure lead to a decreased fitness and/or virulence) (19); (iii) the expectation that neither adverse immune responses nor production of *in vivo* neutralizing antibodies will occur, possibly due to the presence of phages—and their products— among the normal microbiota in humans, and the rapid mode of action that is thought to avoid antibody neutralization *in vivo* (20). Moreover, lysins are amenable to protein engineering strategies (18, 21–24). Typically, the architectural organization of lysins comprises one (or more) enzymatically active domain (EAD) together with a cell wall-binding domain (CWBD) or, sometimes, just an EAD (Fig. 1). Therefore, synthetic biology strategies, such as the construction of completely new lysins made up of different modules as building blocks, have been shown to be achievable. Such strategies enable the design and production of tailor-made antimicrobials, based on the conjunction of diverse functions of interest into a single protein. Functions of interest may include, besides a catalytic activity against the peptidoglycan network (i.e., an antimicrobial activity), increased stability in complex media (25), or, more typically, a certain tropism toward a specific element on the bacterial surface (26) or some other macromolecules, like cellulose (27). The engineering approaches mentioned above have circumvented the alleged inability of lysins to cross the outer membrane (OM) of G− (28, 29). Different kinds of synthetic lysins have been devised to that end. Among them are the so-called artilysins, which are lysins fused to different kinds of membrane-permeabilizing peptides (30), the lysocins, which are lysins fused to elements from bacteriocins that enable bacterial surface recognition and import into the periplasm (22), and the innolysins, lysins fused to phage receptor-binding proteins (31).

However, some lysins also exhibit intrinsic bactericidal activity on G− (32–34). This activity was first noticed for the T4 phage lysozyme (35) and several Pseudomonas aeruginosa phage lysins (36). This unexpected property was attributed to nonenzymatic mechanisms, previously observed in partially denatured hen egg white lysozyme (37), and relies on the presence of antimicrobial peptide (AMP)-like subdomains within such lysins, usually at a C-terminal position (32, 38). Recently, it has been suggested that such AMP-like elements are widespread among lysins from phages infecting G− and that they might cooperate in host lysis by providing an additional affinity for the OM, because of their high net charge in comparison with the negatively charged lipopolysaccharides, and might play a role in lysis itself by exerting a membrane-destabilizing effect (28, 33, 39–41). However, how widespread this trait is has not been yet properly examined, and therefore, its true functional and evolutionary implications are largely unknown. Of note, such AMP-like elements have been successfully used to design AMPs that are active on their own (36, 39, 42).

To uncover the actual evolutionary relevance of these AMP-like elements, as well as other lysin features, such as their domain architecture, in this work, a bioinformatic approach examining a wide collection of lysins was proposed. There are several precedents for the application of homology-based analysis of putative lysin sequences that have paved the way to the systemic comprehension of the coevolution of phage lysins and their hosts (13, 43). The present study aimed to update the picture with the latest available information, as well as to provide answers to the recent questions brought forward by the literature regarding lysin engineering. Therefore, based on current knowledge and available genomic data, we have constructed and curated a comprehensive database of phage lysin sequences. Subsequent analyses on the data included (i) an initial exploration of the database composition, (ii) a cross-reference of information added to the database to check for differential distribution of distinct domain families and their architectural combinations across different bacterial groups, and (iii) an overview of easily computable physicochemical properties (net charge, hydrophobicity, etc.) along amino acid (aa) sequences to explore widespread, relevant differences between groups. The conclusions presented here will strengthen our understanding of lysins’ specificity and variability and help in future drug design efforts based on phage products.

## RESULTS AND DISCUSSION

### Outline.

A total of 9,539 genomes were prospectively obtained from the National Center for Biotechnology Information (NCBI) database (retrieved on April 2020). After a careful curation process (for details, see Materials and Methods), the final database contained 2,182 proteins and a total of 3,303 Pfam (PF) hits (Table 1). Each of these sequences was associated with a bacterial genus corresponding to its described host, for which data on its Gram group and peptidoglycan chemotype were added (Table 1). In total, our database comprised phage lysins from 47 bacterial genera, accounting for up to a total of 2,179 sequences, plus three lysin sequences from PRD1-like phages that infect several enterobacteria. Taking into account all of the identical sequences, the 2,182 different sequences of our data set correspond to 36,365 entries in the NCBI Reference Sequence database (RefSeq; release 202) (44). It should be mentioned that the screening method is biased by the annotation of the source genomes and by current knowledge about the functional domains present in lysins (i.e., by the present set of Pfam-identified lysin functional domains). Therefore, while the final curated data set will be useful to produce a broad view of our current understanding of phage lysins, generalizations should be interpreted with care, since future works may clarify some of the still-unknown aspects of lysin’s composition and function.

**TABLE 1:** Classifiers assigned to host genera and yield of the curation process

				No. of:			
Host genus or group	Gram group	Outer architecture	Chemotype	Retrieved phage genomes	Genes detected by annotation	Genes after curation (final)	PF hits
*Achromobacter*	−	Diderm (lipopolysaccharide)	A1γ	36	32	10	13
*Acidovorax*	−	Diderm (lipopolysaccharide)	A1γ	3	4	2	2
Acinetobacter	−	Diderm (lipopolysaccharide)	A1γ	139	152	50	57
*Aeromonas*	−	Diderm (lipopolysaccharide)	A1γ	95	77	26	27
*Bacteroides*	−	Diderm (lipopolysaccharide)	A1γ	5	6	3	3
*Burkholderia*	−	Diderm (lipopolysaccharide)	A1γ	72	61	27	36
*Caulobacter*	−	Diderm (lipopolysaccharide)	A1γ	42	58	17	17
*Cellulophaga*	−	Diderm (lipopolysaccharide)	A1γ	52	38	9	9
*Cronobacter*	−	Diderm (lipopolysaccharide)	A1γ	45	64	24	26
Enterobacter	−	Diderm (lipopolysaccharide)	A1γ			1	1
Enterobacteria	−	Diderm (lipopolysaccharide)	A1γ	797	300	3	3
*Enterovibrio*	−	Diderm (lipopolysaccharide)	A1γ			2	2
Escherichia	−	Diderm (lipopolysaccharide)	A1γ	684	745	211	231
*Fusobacterium*	−	Diderm (lipopolysaccharide)	A1γ^*a*^	4	3	3	3
Klebsiella	−	Diderm (lipopolysaccharide)	A1γ	266	259	67	71
*Microcystis*	−	Diderm (lipopolysaccharide)	A1γ	8	8	5	5
Pseudomonas	−	Diderm (lipopolysaccharide)	A1γ	625	485	89	98
*Ralstonia*	−	Diderm (lipopolysaccharide)	A1γ	68	21	14	15
Salmonella	−	Diderm (lipopolysaccharide)	A1γ	414	444	90	93
*Serratia*	−	Diderm (lipopolysaccharide)	A1γ	31	40	13	16
*Shewanella*	−	Diderm (lipopolysaccharide)	A1γ			1	1
*Shigella*	−	Diderm (lipopolysaccharide)	A1γ			3	3
*Sphingobium*	−	Diderm (lipopolysaccharide)	A1γ	2	2	1	2
*Sphingomonas*	−	Diderm (glycosphingolipid)	A1γ	7	2	1	1
*Stenotrophomonas*	−	Diderm (lipopolysaccharide)	A1γ	28	25	6	8
*Vibrio*	−	Diderm (lipopolysaccharide)	A1γ	578	225	76	85
*Yersinia*	−	Diderm (lipopolysaccharide)	A1γ	70	62	16	16
*Arthrobacter*	+	Monoderm	A3α^*b*^	316	400	103	161
*Bacillus*	+	Monoderm	A1α	348	400	150	286
*Bifidobacterium*^*c*^	+	Monoderm	A4α	10	2	1	1
*Clostridioides*	+	Monoderm	A1γ			23	25
*Clostridium*	+	Monoderm	A1γ	109	174	30	43
*Corynebacterium*	+	Diderm (mycolic acid)	A1γ	40	30	16	24
*Cutibacterium*	+	Monoderm	A3γ			65	65
*Enterococcus*	+	Monoderm	A3α (E. faecalis), A4α (E. faecium)^*d*^	127	150	50	77
*Lacticaseibacillus*	+	Monoderm	A4α			13	28
*Lactiplantibacillus*	+	Monoderm	A4α			18	42
*Lactobacillus*	+	Monoderm	A4α	105	133	22	41
*Lactococcus*	+	Monoderm	A3α	388	376	121	144
*Leuconostoc*	+	Monoderm	A3α	35	45	11	16
*Levilactobacillus*	+	Monoderm	A4α			9	14
*Limosilactobacillus*	+	Monoderm	A4α			6	14
*Listeria*	+	Monoderm	A1γ	75	87	17	32
Mycobacterium	+	Diderm (mycolic acid)	A1γ	1,990	3,596	299	374
*Propionibacterium*	+	Monoderm	A3γ	202	214	5	8
*Rhodococcus*	+	Diderm (mycolic acid)	A1γ	70	83	31	70
Staphylococcus	+	Monoderm	A3α	349	456	79	155
Streptococcus	+	Monoderm	A3α	1,064	739	246	688
*Streptomyces*	+	Monoderm	A3γ	240	208	97	151
Total^*e*^				9,539	10,206	2,182	3,303
Gram-negative				4,071	3,113	770	844
Gram-positive				5,468	7,093	1,412	2,459

a Contains lanthionine instead of m-DAP.

b Although there seems to be some variety in peptidoglycan structure among *Arthrobacter*, we assigned the most common chemotype.

c There are several chemotypes within *Bifidobacterium*, but A4α was assigned to the only *Bifidobacterium* host entry in our database (Bifidobacterium thermophilus).

d Phage lysins with unassigned *Enterococcus* host species were included within E. faecalis.

e In the last three rows, data are the sum of the column values (phage genomes, genes detected, and genes after curation) or weighted averages (PF hits per protein).

### General differences among lysins.

**FIG 2:**
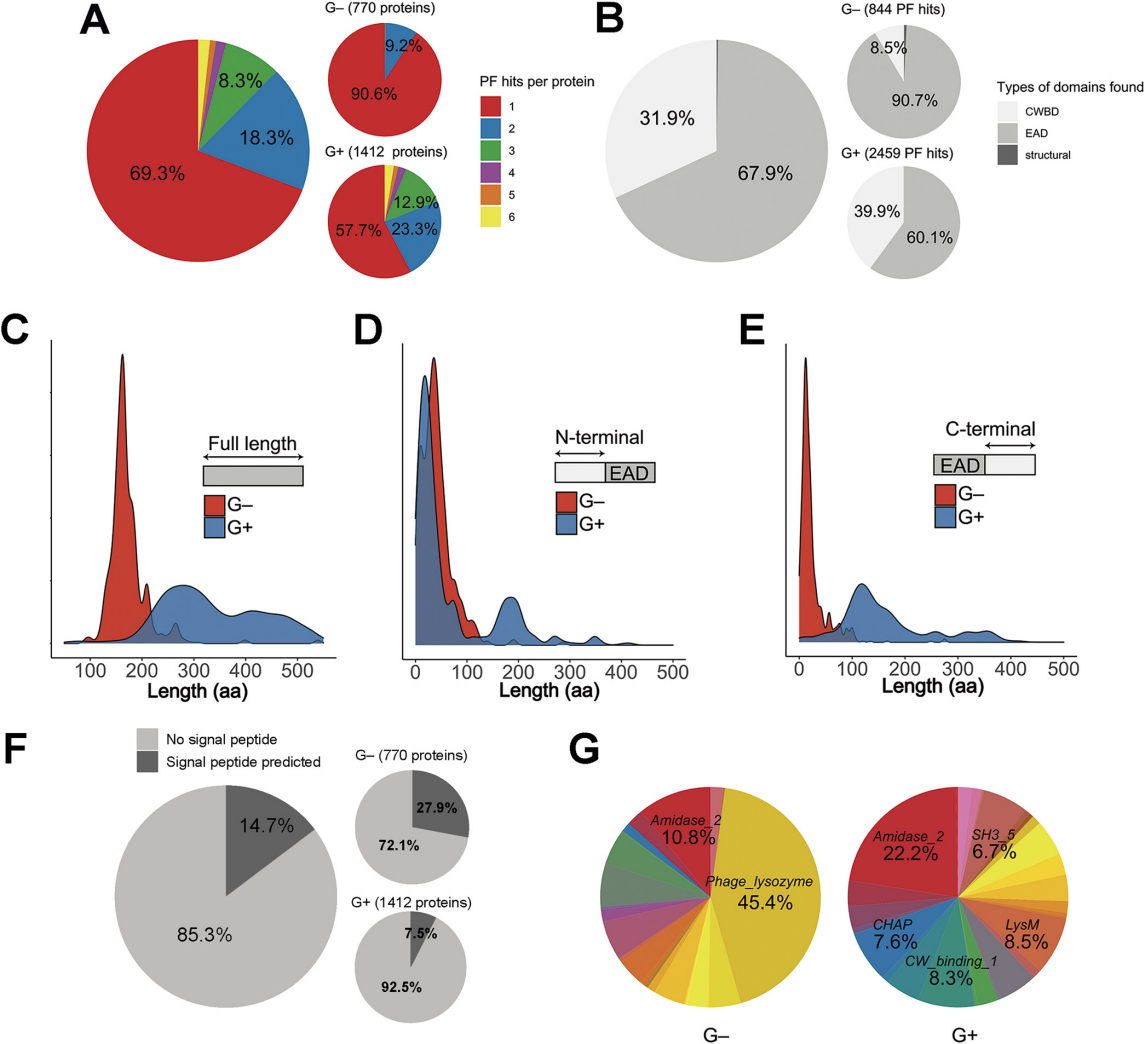
General properties of lysins from phages that infect G+ or G−. (A) Distribution of the number of PF hits predicted per protein. (B) Distribution of domain types. (C) Distribution of protein lengths. (D and E) Distributions of the number of amino acids before (D) or after (E) predicted EADs. (F) Percentage of lysins with a predicted N-terminal signal peptide according to Phobius. (G) PF domain variability (different colors stand for different PF domain families, corresponding to those in Table 2). In distribution charts (C, D, and E), the *y* axis shows an estimation of the distribution density.

For 1,512 of 2,182 sequences (69.3%), only one significant PF hit could be predicted (Fig. 2A). This was especially relevant for lysins from phages infecting G−, given that 90.6% of these proteins were predicted to contain a single functional domain. Nearly 60% of the lysins from phages infecting G+ (for the sake of this work, mycobacteria and their relatives like *Rhodococcus* or *Corynebacterium* were included among G+) harbored only one predicted functional domain. Few lysins appear to contain ≥4 PF hits (Fig. 2A). However, these figures should be considered with caution, since they do not correspond to the number of real functional modules within the protein but to a relatively high number (up to 5) of individual repeats that, together, make up a single functional module. For example, the 37 sequences with 6 PF hits correspond to streptococcal phage lysins having the typical structure [EAD]5×[*CW_binding_1*], with the EAD being either *Amidase_2* (31 hits), *Glyco_hydro_25* (3 hits), or *CHAP* (3 hits) domains. Likewise, not all sequences with a single PF hit should be assumed to contain only a single domain, since many of them might contain other, yet-undefined domains. Also, some repeats (or even full domains) might not be appropriately predicted if there is enough evolutionary sequence divergence. As an example, the domain structure based upon the three-dimensional folding of pneumococcal major autolysin LytA (45) does not concur with the domains predicted by a homology search, since this method is unable to uncover the latest CWBD repeat (Fig. 1A).

As a whole, however, the differential relative amount of single and multiple PF hit sequences between G− and G+ phage lysins (Fig. 2A and B) can be taken into account, in accordance with the usual proposal that G− lysins are typically monomodular, while G+ ones are multimodular (46). Figure 2B shows that some “structural” domains were detected among the lysins from G−-infecting phages. These four entries, bearing domains such as *Gp5_C* or *Gp5_OB*, are probably virion-associated lysins that were kept through the curation process. The mainly monomodular nature of G− lysins is further supported by the evident difference in protein length distributions (Fig. 2C), where G+ phage lysins tend to be larger (median = 317 aa residues) than G− ones (median = 164 aa residues), and also by the differential distribution of sequence lengths before and after the predicted EADs (Fig. 2D and E). Figure 2D shows that EADs from G− phage lysins start at approximately the same point as G+ ones, that is, near the N-terminal end of the protein, except that the EAD starting point distribution is slightly shifted toward the C-terminal part of the enzyme in lysins from G−, perhaps due to the presence, in some cases, of CWBDs at the N terminus (28) or, most likely, because of a high representation of signal anchor release (SAR) lysins among G− phage lysins (Fig. 2F). The latter panel shows that a signal peptide was predicted by Phobius server for 27.9% (215 lysins) of the G− phage lysins, while such elements were predicted for only 7.5% (106 lysins) of the G+ ones. The prediction of a signal peptide is indicative of a SAR mechanism of lysin export into the periplasm (engaging the Sec secretion system) (12). Of note, the G+ EAD starting point distribution shows a secondary local maximum at around coordinate 200. This is consistent with the presence of EADs at a medial location within the protein, something that has already been observed in many G+ phage lysins (13, 43). According to Fig. 2E, most G+ EAD hits have much more “space” at the C-terminal region than G− ones (the respective medians of C-terminal length after EAD hit distributions for G− and G+ are 16 and 136 aa residues). The additional length at the C-terminal part of G+ phage lysins can be occupied by CWBDs or additional EADs, and, taken together, all this evidence supports the common postulate that most detected G− lysins are monomodular.

Finally, Fig. 2G illustrates that, in contrast with the case of G− lysins, G+ lysins present a high diversity of different types of domains. There is a remarkable predominance of the EADs belonging to the *Phage_lysozyme* family of proteins in G− lysins (45.4% of total hits), whereas *Amidase_2*, the most frequent EAD among G+ phage lysins, accounted only for 22.2% of G+ PF hits.

### Differential distribution of domain families among different bacterial host groups.

**FIG 3:**
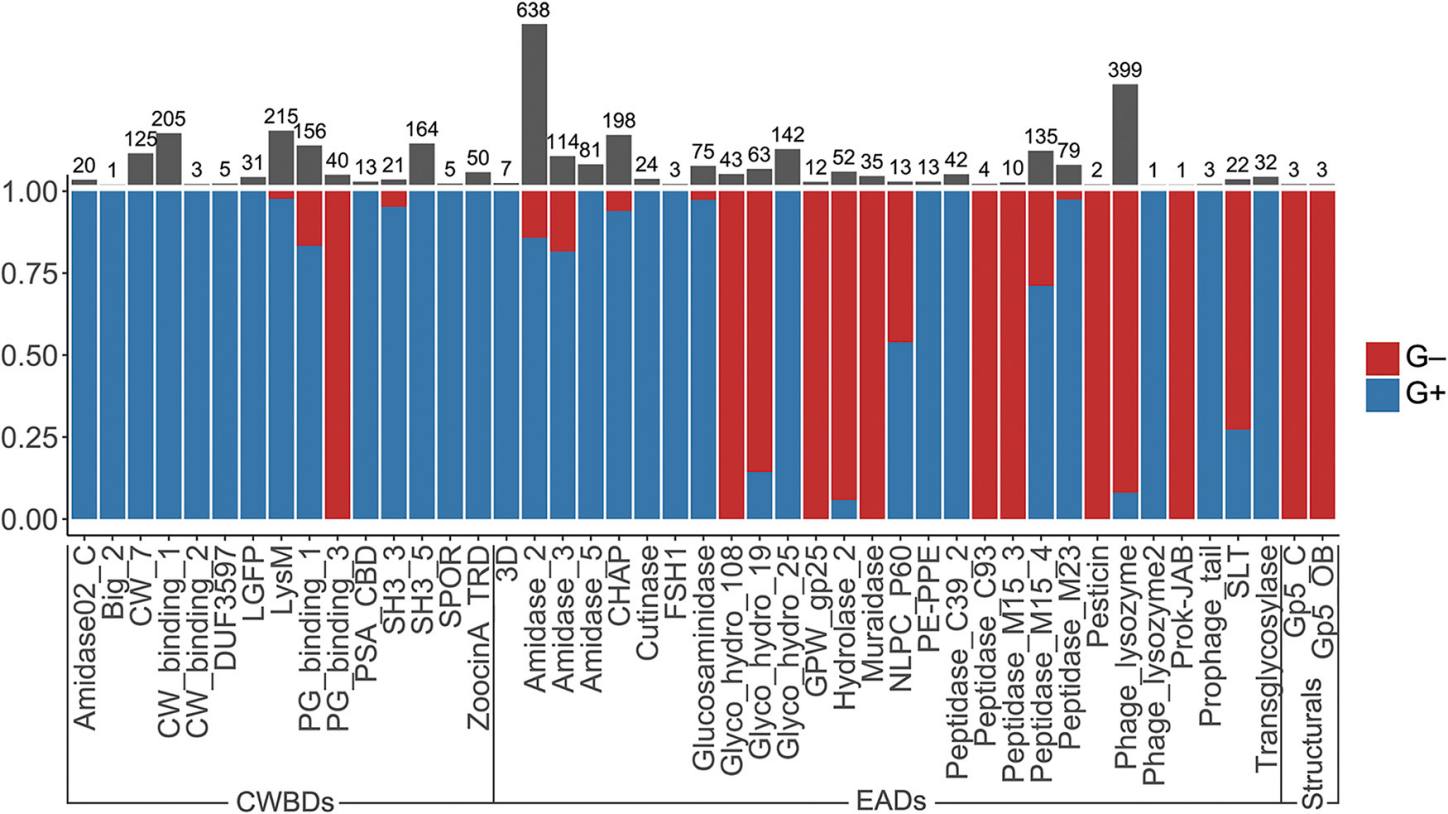
Differential distribution of PF hits among G− and G+ bacterial hosts. The *y* axis shows the proportion of PF hits found in G+ within a given domain family. Gray bars and numbers above the graph represent the total number of hits of each PF domain.

A distribution analysis of each PF family among bacterial hosts was performed (Table 1). Of the total 3,303 PF hits analyzed, 2,460 corresponded to phages infecting G+ bacteria; 2,243 (1,477 G+ and 766 G−), 1,054 (982 G+ and 72 G−), and 6 (G−) corresponded to EADs, CWBDs, and structural domains, respectively (the sources for domain classification as EAD, CWBD, or structural can be found in Table S2 in the supplemental material). When the differential Gram group classification of each PF hit was analyzed, it was found that EADs like *Amidase_5*, *Glyco_hydro_25*, *Peptidase_C39_2*, and *Transglycosylase* were exclusive to G+, whereas *Glyco_hydro_108* and *Muramidase* were characteristic of phages infecting G−. Other EADs like *Amidase_2*, *Amidase_3*, *CHAP*, *Glucosaminidase*, *Peptidase_M15_4*, and *Peptidase_M23*, were common in G+, whereas *Glyco_hydro_19*, *Hydrolase_2*, and *Phage_lysozyme* dominated among G−. In addition, *CW_7*, *CW_binding_1*, *LGFP*, *SH3_5*, and *ZoocinA_TRD* constituted the CWBDs of G+, and although *LysM* and *PG_binding_1* were most frequently found in G+ lysins, they also appeared sometimes among G− lysins (Table 2 and Fig. 3). *PG_binding_3* was the only CWBD exclusive to G− lysins. Interestingly, all of the 40 *PG_binding_3* occurrences were accompanied by *Glyco_hydro_108* at the N-terminal moiety, yielding an architecture ([*Glyco_hydro_108*][*PG_binding_3*]) that was widespread among gammaproteobacteria.

**TABLE 2:**
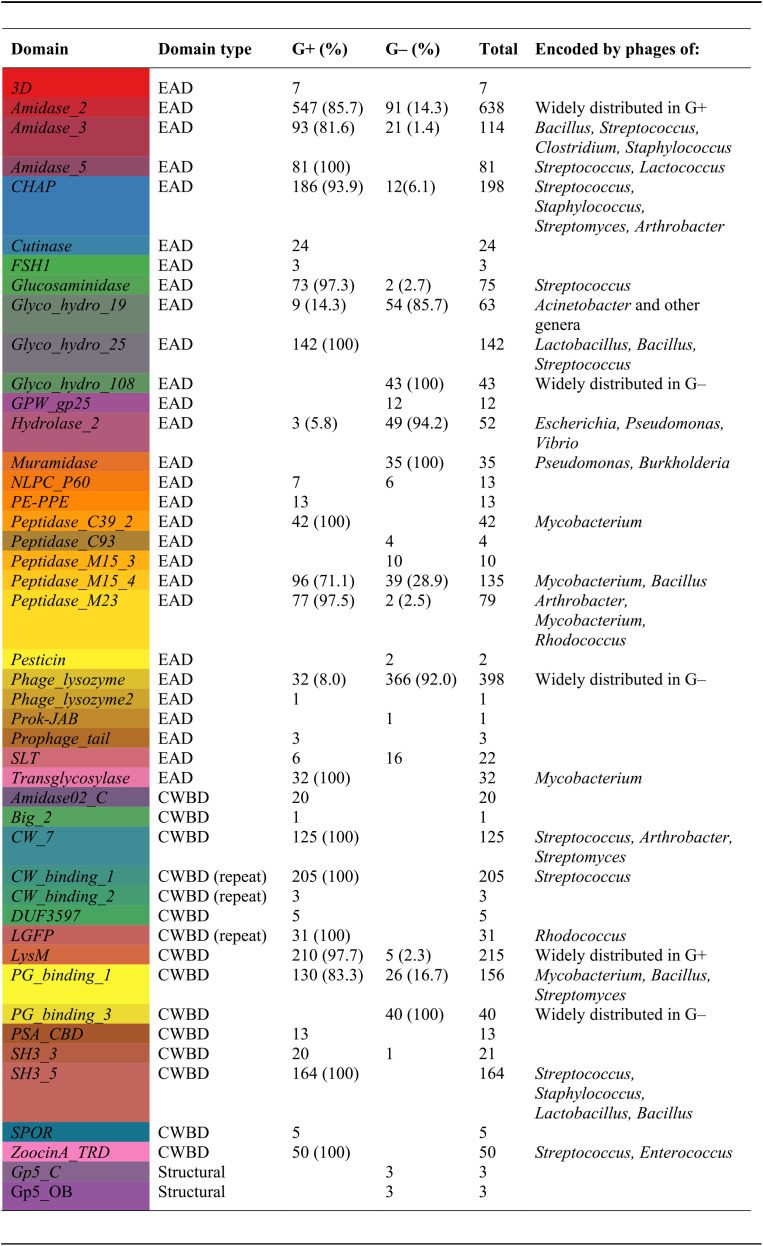
Distribution of PF hits of phage lysins from Gram-positive and Gram-negative bacteria^*a*^

a Percentages and further remarks are shown only for domains represented by at least 30 hits.

**FIG 4:**
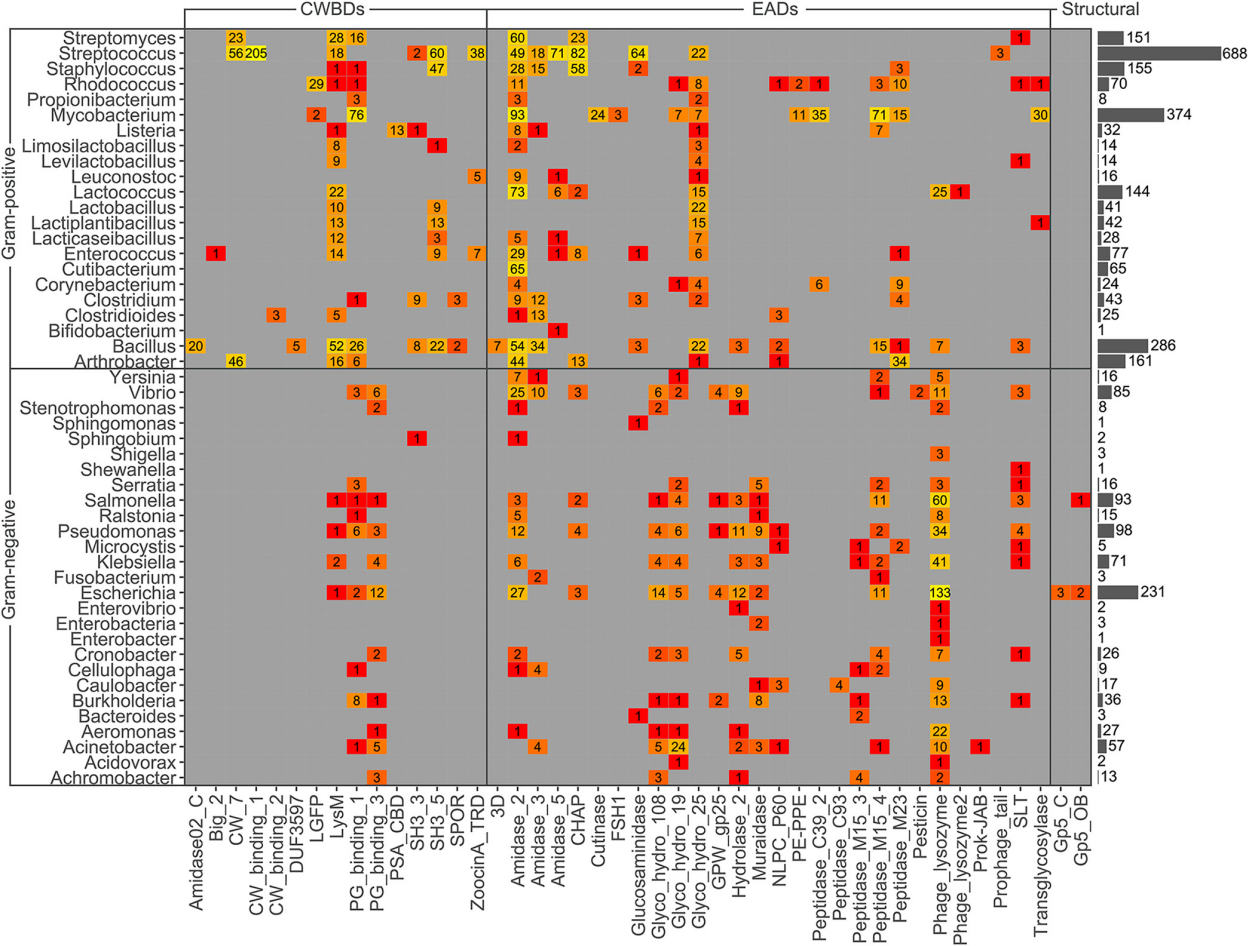
Heat map of PF hits distribution across host bacterium genera. Numbers within each tile indicate the number of hits predicted for the corresponding taxon and PF family. The color scale represents the number of hits from low (red) to high (yellow). Gray bars on the right represent the total number of PF hits predicted within each genus.

**FIG 5:**
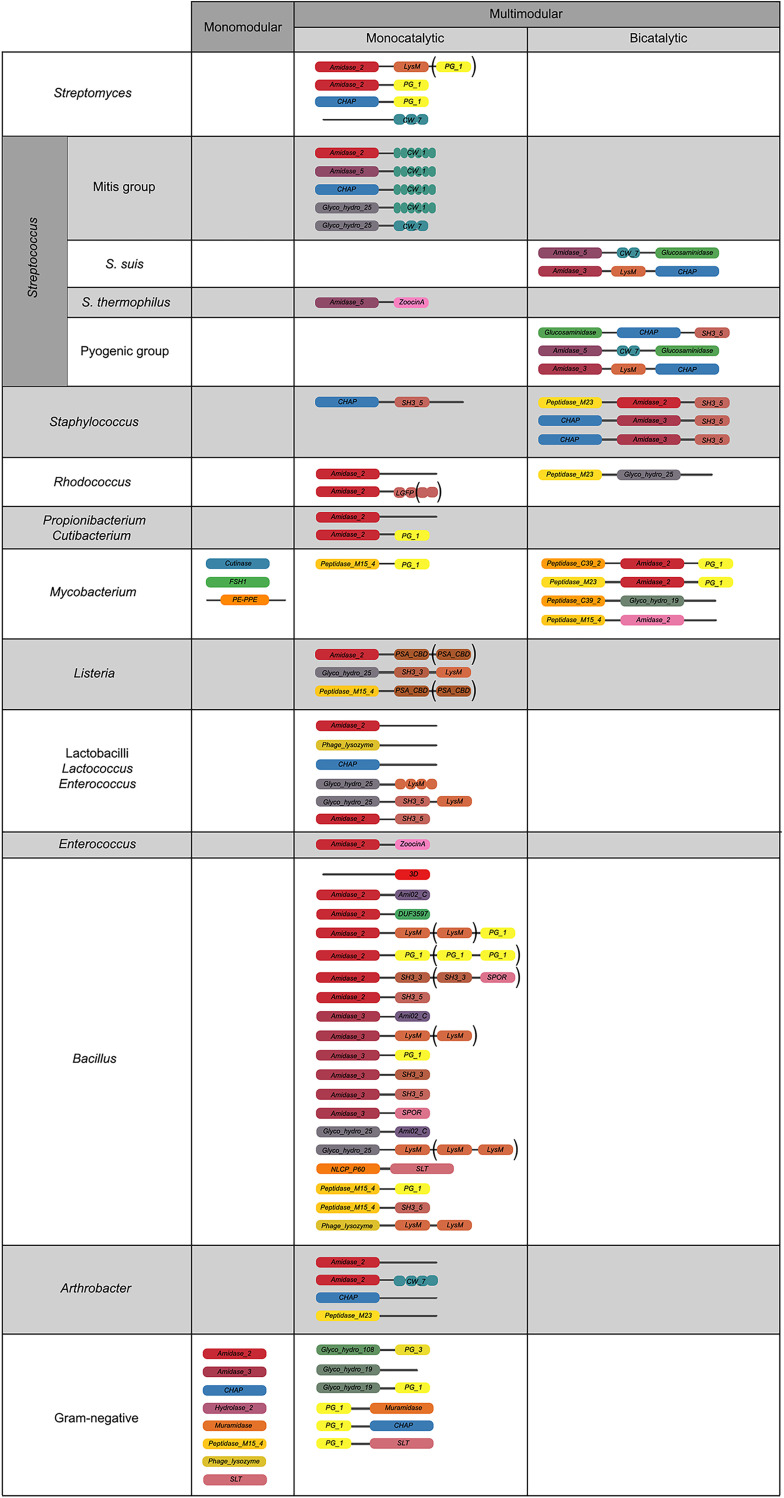
Relevant architectures observed in lysins from phages infecting different taxonomic groups of bacteria. Different colors indicate different domains; brackets denote domains that appear in only some representatives of the depicted architecture.

**FIG 6:**
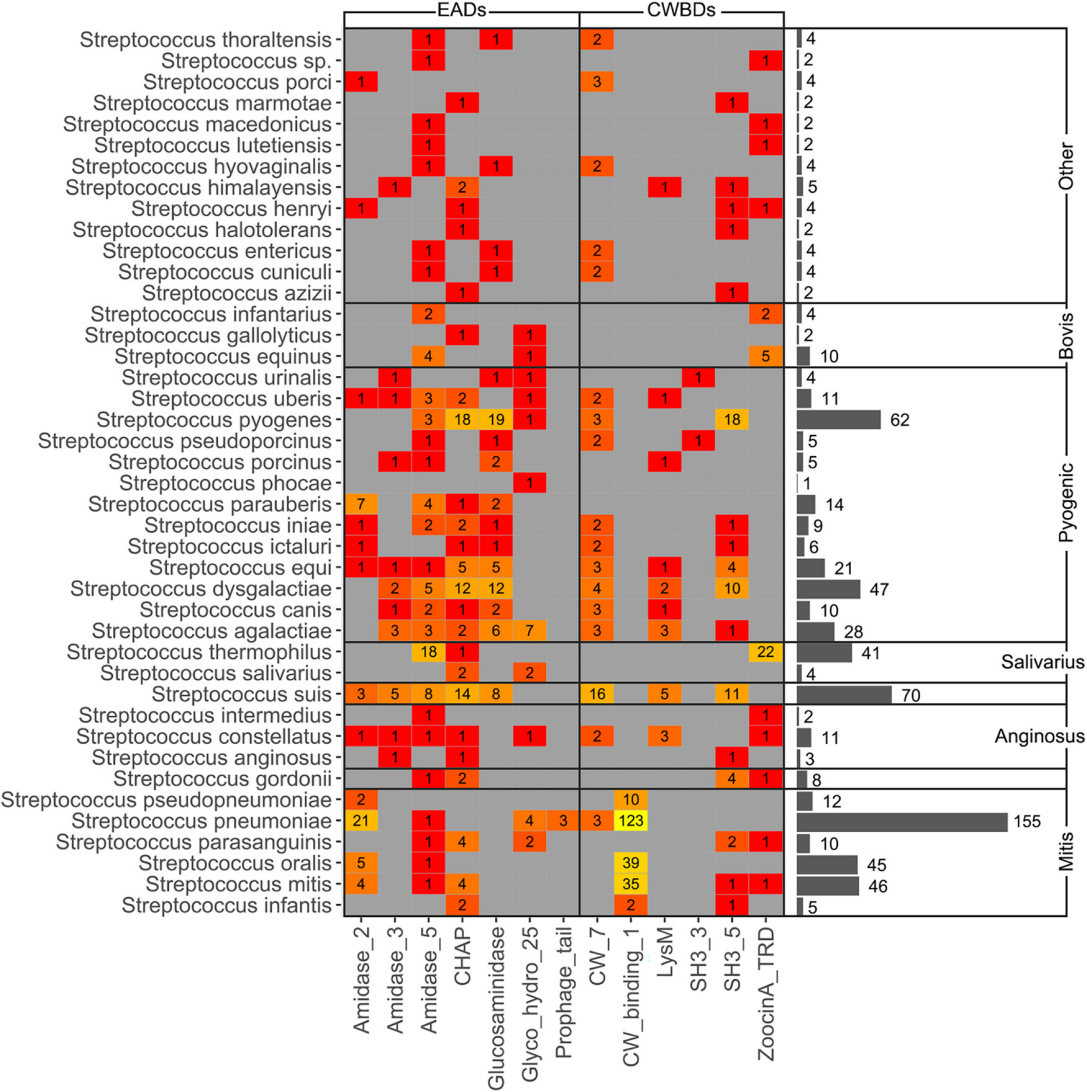
Heat map depicting PF hits distribution among different streptococci. Numbers within each tile indicate the number of hits for the corresponding taxon, whereas colors express a scale from lower (red) to higher (yellow) number of hits. Gray bars show the total number of PF hits for each streptococcal species.

Trends in distribution of PF domains among genera, rather than Gram group, were a bit more complex (Fig. 4 and 5), although some conclusions could be reached. To begin with G+ CWBDs, the *CW_binding_1* repeats were only encoded by phages infecting streptococci, whereas *CW_7* constitutes the CWBD of many phage lysins of Streptococcus, *Arthrobacter*, and *Streptomyces*. *CW_binding_1* repeats are known to bind choline residues present in the teichoic acids of Streptococcus pneumoniae and its relatives (i.e., streptococci of the mitis group) (47, 48) and therefore appeared only within our data set among this group of bacterial hosts (Fig. 6). *CW_7* repeats are known to bind a conserved peptidoglycan motif and are thus less restricted in the variety of bacteria they may recognize (49). *LysM* domains were also widely distributed in G+, *ZoocinA_TRD* was very common among Streptococcus thermophilus, and *PSA_CBD* was exclusive for *Listeria* phage lysins. As for EADs, *Amidase_5* was very frequently found among streptococci and *Amidase_2* was generally abundant among all G+.

Another exclusive trait of some G+ lysins was the concurrence of two distinct EADs. This was observed for phage lysins from Streptococcus suis, pyogenic-group streptococci, staphylococci, and mycobacteria. A possible explanation for multicatalytic lysins is an increased lytic efficiency over monocatalytic ones, since activities attacking different sites of the peptidoglycan are known to act synergistically in peptidoglycan degradation (50). Such synergy could also imply a decreased chance for the appearance of resistant peptidoglycan mutants (51). It has also been shown that the synergistic concurrence of both activities is sometimes needed for full activity. Thus, it has been suggested that some phages may have evolved a regulatory mechanism to avoid the lysis of other potential host cells relying on the proteolysis of bicatalytic lysins by host cell proteases. Then, both EADs would be disjointed by proteolysis upon host cell lysis, and the degraded lysins would no longer be active against the nearby bacterial population (52). This should be especially relevant for phages infecting G+, which lack a protective OM hindering the lysis of other bacterial cells from without, and this accounts for the exclusiveness of the bicatalytic architecture among phages infecting G+. In some other cases, however, it is the high affinity of the CWBD that has been proposed as the mechanism that maintains lysins tightly bound to cell debris, preventing widespread lysis of the bacterial community (53), which is also an argument for the widespread presence of CWBDs among G+ and not among G−. Additionally, it has also been proposed that, at least in some cases, the central domain predicted to be an EAD may in fact have an auxiliary role in substrate binding, rather than being an actual EAD (54).

Staphylococcal phage lysins presented reduced EAD variability, normally using *Amidase_2*, *Amidase_3*, and/or *CHAP* domains, with *SH3B_5* being the preferred CWBD, in agreement with previous results (55). In some cases, the staphylococcal *SH3B_5* has been shown to bind the peptidoglycan with the characteristic pentaglycine interpeptidic bridge of Staphylococcus (56). Domains putatively assigned an esterase activity (*Cutinase*, *FSH1*, and *PE-PPE*) were present only in phages from Mycobacterium and its relatives, presumably as type B lysins. The *LGFP* repeats, quite common among *Rhodococcus* phages, might be a specific CWBD among such *Corynebacteriales*. Peptidase EADs were common and diverse among mycobacteriophages, in contrast with other G+ phages, which do not typically contain peptidase EADs other than *CHAP*. Of note, *CHAP* domains have been described sometimes as peptidases but on other occasions as *N*-acetylmuramoyl-l-alanine amidases (NAM-amidases) (57, 58).

Regarding G−, the most widely spread architecture of G− phage lysins was monomodular, harboring a single *Phage_lysozyme* domain, which accounted for half (50.8%) of the identified G− lysins in our database. Another architecture that was only found in G− lysins is the localization of a CWBD at the N-terminal end (for example, [*PG_binding_1*] [*Muramidase*]), although it was not at this position in every case (e.g., the architecture [*Glyco_hydro_108*] [*PG_binding_3*] was also present).

**FIG 7:**
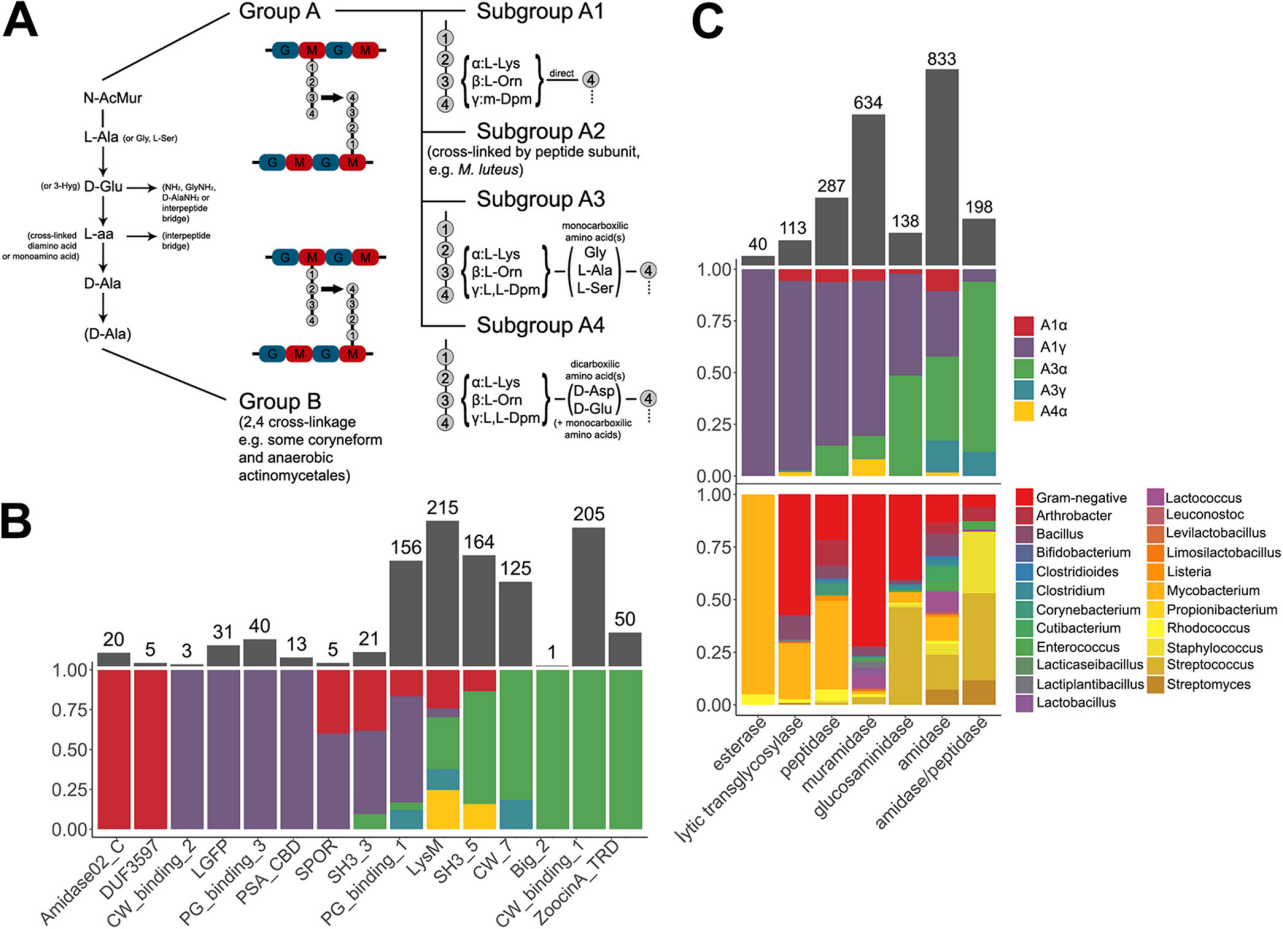
Differential distribution of CWBDs and catalytic activities across peptidoglycan chemotypes and taxonomic groups of bacterial hosts. (A) Schematic representation of the relevant peptidoglycan chemotypes present for the bacterial hosts in our data set. (B) Distribution of CWBD PF hits among chemotypes. (C) Distribution of catalytic activities of EAD PF hits among chemotypes and taxonomic groups.

**FIG 8:**
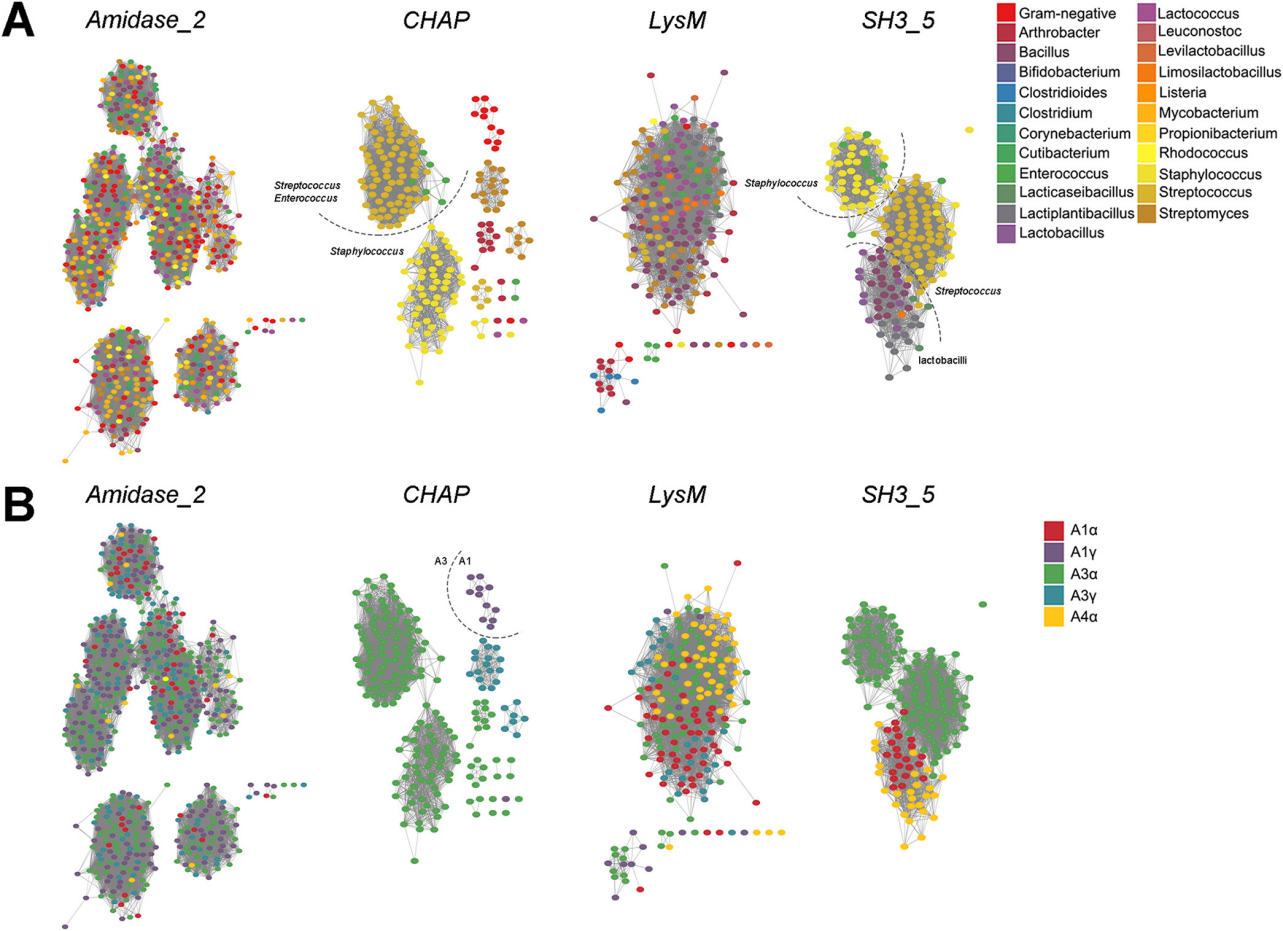
SSNs of the PF hits in our data set corresponding to different domain families. (A) Classification of sequences according to the taxonomic group of the corresponding bacterial host. (B) Classification by peptidoglycan chemotype of the host. Each node represents a single sequence. Dashed lines separate recognizable similarity clusters. The edge similarity cutoff was ≈ 40% for *CHAP*, *LysM*, and *SH3_5* and ≈ 30% for *Amidase_2*.

The correlation between domain distribution and peptidoglycan composition might also shed some light on the relationships of different domain families with different taxa. To that end, the chemotypes classification of peptidoglycan proposed by Schleifer and Kandler (59) was used (Table 1). Briefly, such classification hierarchically relies on (i) the site of cross-linkage of the peptide subunit of the peptidoglycan, (ii) the nature of the cross-link, and (iii) the specific residue at position 3 within such peptide subunit (Fig. 7A). Starting with CWBDs (Fig. 7B), classification by chemotype did not provide a better explanation for specificity than other genus-specific traits, as discussed above. However, some specificities could be found (e.g., *Amidase02_C* appears only in phages that infect A1α bacteria or *PG_binding_3* only in A1γ), and some CWBDs that are widespread among different chemotypes could also be observed (*PG_binding_1*, *LysM*, and *SH3_5*). In general, however, it cannot be stated that peptidoglycan composition is a major determinant for CWBD specificity, except for some cases, such as *ZoocinA_TRD* domains, which have been proposed to bind A3α with two Ala residues at the cross-link (60). The poor performance of chemotype as an *a priori* predictor of the CWBD PF family ligand is more evident if we consider the CWBD types which appeared widespread among many different chemotypes, such as *LysM* and *SH3_5*. To check whether this apparent “promiscuity” may be linked to the presence of subfamilies with potentially different ligands or if it could rather be a true promiscuous binding, sequence similarity networks (SSNs) were constructed with the PF hits of *LysM* and *SH3_5* (Fig. 8). The *LysM* SSNs did not show prominent similarity clusters classified either by taxon or by chemotype of the bacterial host. This suggests that *LysM* could be a truly universal CWBD that would bind to a conserved cell wall ligand. The rather generic description of *LysM* ligands in the literature (as *N*-acetylglucosamine-containing polysaccharides) is in agreement with this observation. *SH3_5*, however, displayed at least two differentiated sequence similarity groups that correlated rather well with different taxonomic groups (namely, staphylococci versus streptococci and lactobacilli). In fact, the literature shows that while lytic enzymes with predicted *SH3_5* domains typically recognize polysaccharides (and peptidoglycan in particular), there seem to be different specializations. For example, the CWBD of the Lactiplantibacillus plantarum major autolysin binds many different peptidoglycans with low affinity, with glucosamine being the minimal binding motif (61), while *SH3_5* domains from staphylolytic enzymes have been shown to be rather specific to cross-linked peptidoglycans (like the A3α peptidoglycan of Staphylococcus and Streptococcus) and that the nature of the cross-link itself determines the affinity of such CWBDs for the peptidoglycan (56, 62).

Additional information could be drawn from this analysis when it was applied to the different catalytic activities detected (Fig. 7C). First of all, NAM-amidases were the most represented type of domains and also those that appeared among more different taxonomic groups and chemotypes, even more so than lysozymes. Indeed, *Amidase_2*, the most abundant PF domain in our data set (638 hits), appeared in lysins from both G+ and G− phages. The SSN in Fig. 8 shows, however, that although *Amidase_2* seems a rather diverse group, with various observable similarity clusters, none of these clusters correlates with any of the classifiers of the bacterial hosts tested.

Muramidases were quite overrepresented among G− phages (chemotype A1γ) because of the widespread presence of *Phage_lysozyme* domains. Glucosaminidases appeared evenly both against A1 and A3 peptidoglycans, but whereas in G+ phages (which comprise all A3s and a few A1s) glucosaminidase activity was represented by *Glucosaminidase* PF domain, the only domain putatively assigned with a glucosaminidase activity among G− phages was *Glyco_hydro_19* (Fig. 4 and 7).

Another interesting observation is that peptidase activities were more common among lysins from phages infecting bacteria with subgroup A1 peptidoglycans, which, in turn, display the simplest cross-linkage of all types, lacking an interpeptide bridge. Thus, peptidases were not uncommon among G− phages and were also present in phages from A1 chemotype G+ (especially mycobacteriophages but also listeriophages and phages from *Clostridium*, *Bacillus*, or *Corynebacterium*). On the other hand, amidase/peptidases, which is the label given to *CHAP* domains (Table S2 in the supplemental material), were much more prevalent among A3 G+ and only seldom present in lysins from phages infecting A1 bacteria (namely, some G−). This suggests that if there is an A3-specific peptidase activity, it would be the one located in *CHAP* domains. It makes sense that different peptidase structures have evolved toward A1 and A3 peptidoglycans, since the complexity of their peptidoglycan peptide moieties differs significantly. Adding to this conclusion, the *CHAP* SSN (Fig. 8) did show a similarity clustering of the few *CHAP* examples in lysins from A1 phages, besides an apparent differentiation of Staphylococcus and Streptococcus/*Enterococcus*.

### Physicochemical analysis of phage lysins from Gram-positive versus those from Gram-negative bacteria.

**FIG 9:**
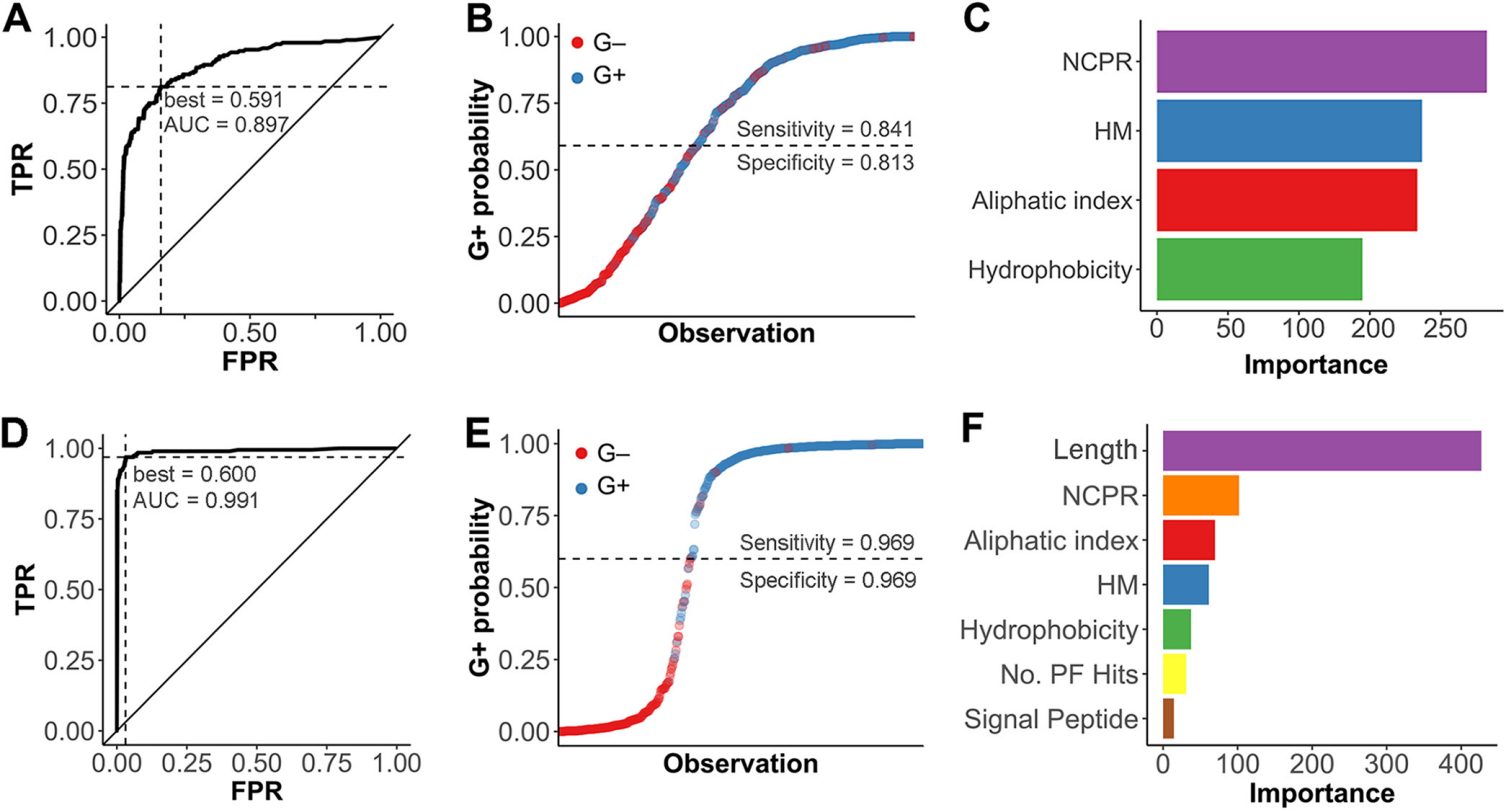
Random forest prediction and classification of Gram groups of bacterial hosts based on physicochemical properties of lysins (A, B, and C) or on those properties plus others relative to lysin architecture (D, E, and F). (A and D) ROC curves of the random forest predictive models (TPR, true-positive rate; FPR, false-positive rate). ROC best points of positive-group (G+) probability for outcome maximization are presented, as well as the AUCs. (B and E) Random forest castings of bacterial host Gram group on the testing subset of lysin sequences. The dashed lines represent the G+ probability threshold for classification based on the respective ROC best points. (C and F) Importance (i.e., mean Gini index decrease for each variable) of each of the four descriptors used for classification within each model. HM, hydrophobic moment.

The results analyzed so far support a distinct distribution of domain architectures and families among lysins that infect different kinds of bacteria and even hint at an association of a differential distribution with some cell wall properties. To check whether such variations can also be correlated with a measurable difference in physicochemical properties, net charge, net charge per residue (NCPR), hydrophobicity, average hydrophobic moment, and aliphatic index were calculated and used to implement a random forest (Fig. 9). This way, the aforementioned physicochemical variables were used as classifiers for the prediction of the host bacterium Gram group of lysins. The resulting algorithm yielded a receiver operating characteristic (ROC) plot with an area under the curve (AUC) of 0.897, which can be interpreted as a good predictive ability (Fig. 9A). Using the probability threshold (0.591) derived from the best point of the ROC curve (which maximizes true-positive rate and minimizes false-positive rate), G+/G− classification upon the testing subset (Fig. 9B) yielded an accuracy of 87.9% with sensitivity and specificity of 84.1% and 81.3%, respectively (with the classification as G+ being the “positive” one). According to the subsequent analysis (Fig. 9C), NCPR was the most relevant variable to distinguish between G+ and G−, followed by average hydrophobic moment, aliphatic index, and, finally, hydrophobicity. In general, these results suggest that lysins from phages that infect G+ and G− can in fact be differentiated by their physicochemical properties in a relatively efficient manner. Nonetheless, a second random forest model constructed with additional variables (namely, the protein length, number of PF hits, and the prediction of N-terminal signal peptides) rendered a better result in classifying lysins (96.15% accuracy) (Fig. 9D to F). In the latter case, the variable with a greater impact in classification was the protein length (Fig. 9F), which is not surprising given the data presented in Fig. 2C, followed by the physicochemical properties. These results prove that it is possible to *a priori* predict the Gram group of the bacterial host of a given lysin with rather high accuracy and also that the physicochemical features of the lysins do play a role in the differentiation of such classes.

**FIG 10:**
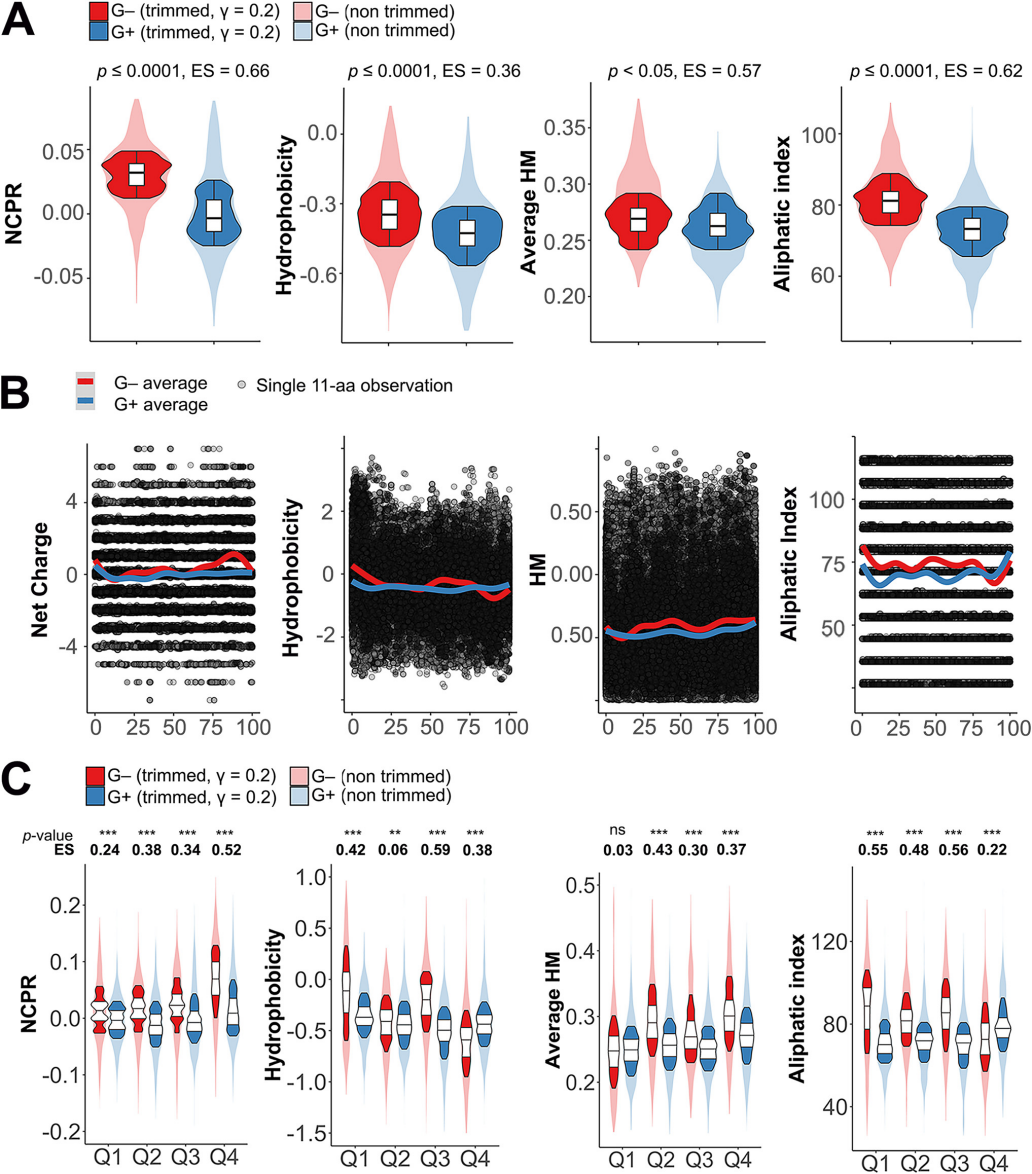
Differential physicochemical properties distribution among G+ and G− phage lysins. (A) Distribution of net properties calculated along the whole protein sequences of lysins from phages infecting G− or G+. (B) Local computation of physicochemical properties. Each dot represents the particular value calculated for an 11-aa window in a given lysin. Continuous lines are average tendencies based on either all G− or all G+ data points. (C) Distribution of different properties at quartiles of lysin sequences. Asterisks indicate *P* values (**, *P* ≤ 0.01; ***, *P* ≤ 0.001) obtained from the Yuen-Welch test for trimmed means with a trimming level (γ) of 0.2. ES indicates the Wilcox and Tian ζ measurement of effect size.

Indeed, the net charge distribution (normalized by protein length) was significantly higher in G− lysins than in G+ ones (*P ≤ *0.0001; effect size [ES] = 0.66) (Fig. 10A, leftmost panel). Moreover, the average prediction of local net charge suggested that this difference is located mainly at the C-terminal part of G− lysins (Fig. 10B). A more thorough comparison (Fig. 10C) seemed to confirm this. At every sequence quartile of the proteins (i.e., contiguous fragments of sequence with a length equal to 1/4 of the total number of amino acid residues in the original protein sequence), the net charge distribution of G− lysins showed a significantly higher net charge. However, the actual size of this shift was only moderate along the sequences (ES between 0.24 and 0.34), but it was, again, higher at the final quartile (ES = 0.52).

Hydrophobicity was also higher in G− lysins, but the difference relative to that of G+ ones is smaller (ES = 0.36). This might be related to the rather inconsistent pattern shown by average local hydrophobicity and sequence quartile comparison (Fig. 10B and C). G− lysins tended to have a more hydrophobic N-terminal region, which could be correlated with the greater abundance of predicted signal peptides among the G− group mentioned above (Fig. 2F). At the C-terminal moiety, the tendency was reversed, which can be explained by the relative abundance of positively charged residues shown before for G− phages. It is at the third quartile (Q3), immediately before the high positive net charge patch described above, where the difference was statistically more relevant (*P* ≤ 0.001; ES = 0.59), with higher values in G− phages. There was also a statistically significant difference in the average hydrophobic moment distributions between G+ and G− phage lysins. For the G− group, the local plot (Fig. 10B) showed a higher tendency to present greater hydrophobic moments along the whole protein length except for the N-terminal part. Analysis of sequence quartiles confirmed a statistically significant superiority of average hydrophobic moment for G− phage lysins except at the N terminus. The aliphatic index was also significantly higher in G− phage lysins, although G+ phage lysins showed an aliphatic index peak at their C-terminal regions that surpassed that of G− phage lysins (coincidental with the G− basic amino acid peak, which, understandably, would lower both hydrophobicity and aliphatic index at Q4) (Fig. 10C).

Taking all these observations together with the results yielded by the random forest prediction, we can conclude that the physicochemical difference between lysins from phages that infect G+ or G− is manifested as a higher positive net charge of G− phage lysins, particularly at the C-terminal end, combined with a greater propensity for incorporating aliphatic amino acids, and likely results in amphiphilic structures.

**FIG 11:**
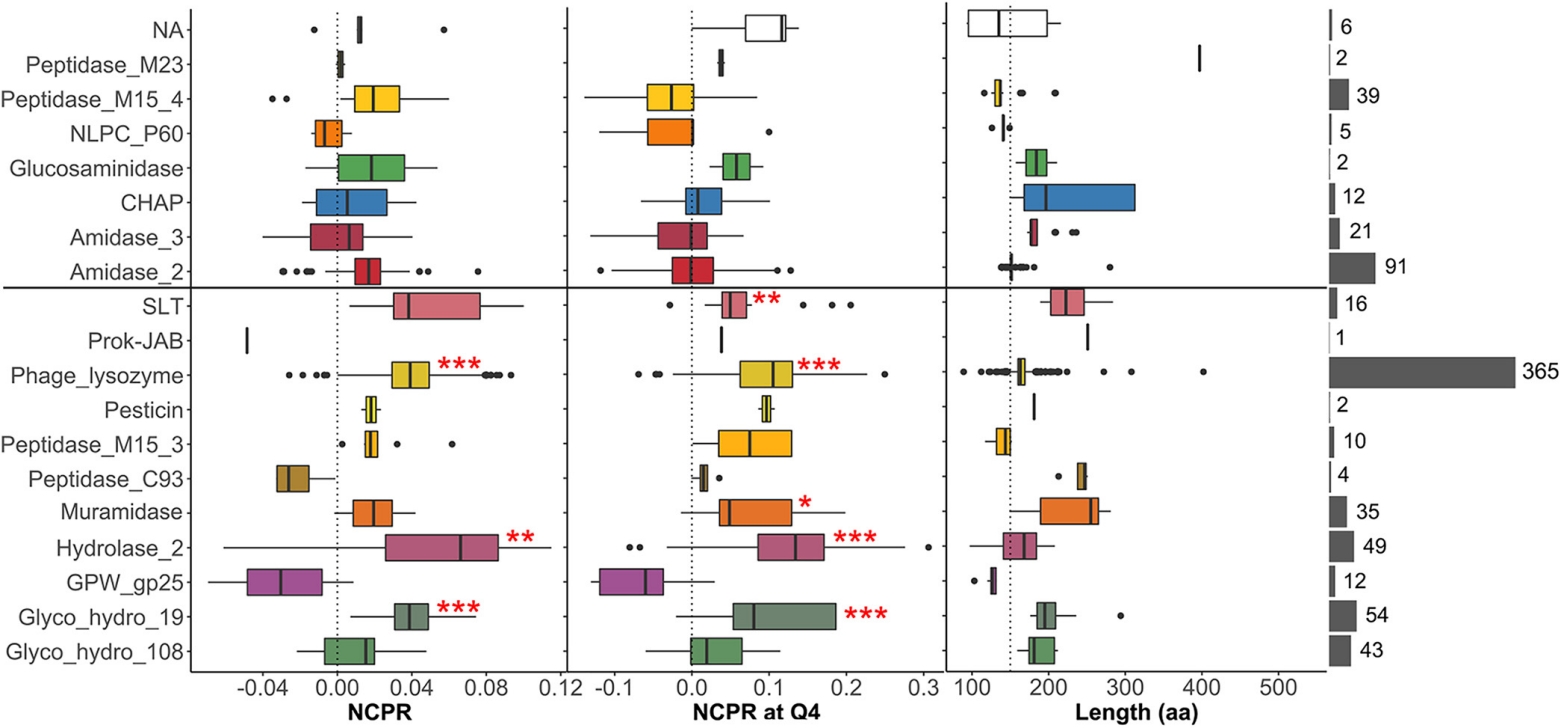
Net charge distribution of lysins from G−-infecting phages classified according to the predicted EAD. The rightmost gray bars depict the number of lysins classified into each EAD group (lysins within the NA group are those for which an EAD was not assigned). All groups were compared with the distribution of the *Amidase_2* domain, as a highly represented, near-neutral control using Welch’s test on γ = 0.2 trimmed means with *post hoc* Bonferroni correction (*, *P* ≤ 0.05; **, *P* ≤ 0.01; ***, *P* ≤ 0.001).

**FIG 12:**
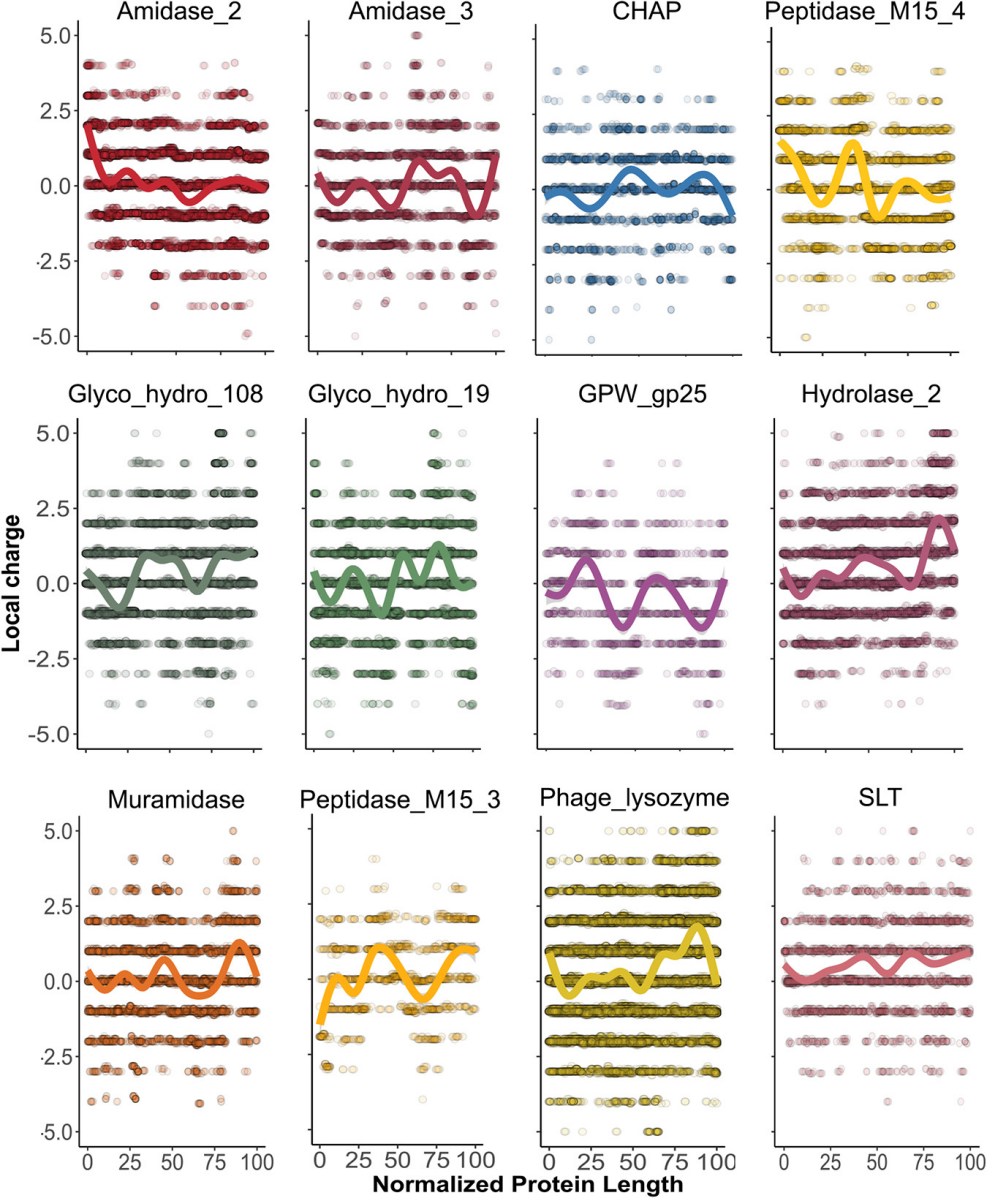
Local computation of physicochemical properties in lysins from G− infecting phages classified according to EAD predictions. Each dot represents the particular value predicted for an 11-aa window from a given lysin. Continuous lines are average tendency lines.

A closer examination of net charge (and C-terminal net charge) of lysins from G−-infecting phages indicated that the high positive patch trait seems specific to some domain families. As a whole, a statistically significant higher NCPR value was found in lysins bearing *Phage_lysozyme*, *Hydrolase_2*, and *Glyco_hydro_19* domain families (Fig. 11). At the C-terminal part, higher NCPR was found in lysins bearing the domains mentioned above but also in *SLT* and *Muramidase*. The average local net charge tendency showed for each EAD group (Fig. 12) confirmed that a local high-positive-charge peak appears in the protein region immediately before the C-terminal apex.

Interestingly, all of the aforementioned domains that present a high-positive-charge patch at their C-terminal region were preferentially present in lysins from phages that infect G− (Table 2). This observation provides a basis to argue an evolutionary tendency in some G− infecting phages to develop AMP-like subdomains at the C-terminal moiety of their lysins. Indeed, such subdomains contain features typical of AMPs (such as the high net charge accompanied by a high local hydrophobic moment, hydrophobic patches, etc.) and may play a role in the interaction between lysins and cell wall in G− bacteria. Electrostatic interactions may play a significant role in phage-bacterium interplay, as suggested for modular lysins from phages that infect G+ bacteria. For example, it has been shown that the negative net charge of many G+ lysins hinders their ability to approach the negatively charged cell wall from without (18, 63). This renders the affinity-based interaction of the CWBDs with their cell wall ligands essential for lysin activity, at least when they are exogenously added. The essentiality of CWBDs has been shown for several lysins (20) and, as is the case for other carbohydrate-active enzymes, can be related to the insoluble nature of the substrate. Therefore, the CWBD serves the purpose of providing affinity for the nondiffusible substrate (in this case, the peptidoglycan). Once again, though, generalizations should be made with caution, because there are also cases reported of single catalytic domains that lysed G+ cells from without more efficiently when their CWBD was removed (64). On the other hand, and to our knowledge, there are only a few cases reported in which CWBDs appear to increase the efficiency of cell wall-lysin interaction in G− lysins (21).

We have already shown, based on our own data, that it is safe to say that G− lysins are monomodular. Thus, taking this theoretical framework into account, it could be argued that some G− lysins could have evolved a distinct strategy to grant cell envelope interaction, namely, the AMP-like regions, rather than containing an additional CWBD, which, incidentally, might be essential for postlytic regulation in G+ but not in G− (53). The AMP-like subdomains, besides providing better anchorage to bacterial surface structures, might also act as an additional mechanism for effective lysis of G− bacteria (i.e., a spanin-like function), and it is even conceivable that the AMP-like elements could take over a holin function, since there are examples of lysins able to gain access to the peptidoglycan on its own, without the necessary co-occurrence of a holin (32, 65). There are indeed abundant examples in literature of the ability of G− lysins to interact with bacterial membranes and permeabilize them (33, 38, 39, 66), a trait that, it is plausible to say from both our own analysis and the experimental results of many works, would reside in such AMP-like elements.

It was recently suggested that phages lacking spanins (spaninless phages) need additional gene products that behave similarly to AMPs to induce efficient host cell lysis (67). This hypothesis could also be extrapolated to the presence of AMP-like membrane-permeabilizing subdomains, which could then compensate for a missing spanin system. However, the lack of extensive evidence on the latter issue warns against this kind of generalizations for the time being. If we assume that AMP-like subdomains behave as membrane-active molecules, the identification of these elements within lysins could also provide a way to predict the ability of such lysins to better interact with the OM from without, and thus their antimicrobial potential. This kind of approach has already been successfully tested at least once, as demonstrated, for example, in the work of Larpin et al. (68), in which AMP-like subdomain bearing lysins were screened by means of a homology search based on a previously described AMP (39).

### Concluding remarks.

Phages and their bacterial hosts are constantly evolving in a codependent manner (69). From the point of view of phage lysins, this means that such molecules have adapted to the particular structures and features of the host cells. This adaptation can be described as the functional adjustment of the protein elements to optimally fulfil their purposes: the efficient and regulated degradation of the peptidoglycan. Therefore, lysin structures and cell wall structures must be closely correlated. A way of testing and understanding this relationship is the sequence-based classification of the constituent domains of phage lysins presented here and the analysis of their distribution among (pseudo)taxonomical and structural classes of bacterial hosts. Our procedure yielded several important associations of lysins and cell wall architectures, which can be explained in a structural-functional way.

(i) The first lies in the architectural differences between lysins from phages that infect G+ and G−. Those from G− phages are usually monomodular, whereas lysins from G+-infecting phages are multimodular. Moreover, the bicatalytic type of modular structure appears only among G+. Some possible explanations for this architecture are the requirement for tighter postlytic regulation in G+ and/or a more efficient lytic activity relying on tighter substrate binding or on the synergistic effect of combining different catalytic activities.

(ii) Second is the association of CWBDs with specific bacterial host genera in our data set, together with the literature showing that many of these CWBDs are able to recognize ligands that are specific traits of the related bacterial hosts, for example, *SH3_5* in staphylococcal phages, *CW_binding_1* in Streptococcus mitis group phages, *PSA_CBD* in listeriophages, and *PG_binding_3* in G−. This also manifests the genetic trading between host and parasite, since many of those CWBDs, as well as their bacterial ligands, are also used by the bacterial host surface proteins.

(iii) The third feature is the differential appearance of EAD families within phages that infect bacteria with a certain chemotype, which suggests an adaptation of the enzyme to the structure of the specific peptidoglycan it has to degrade. This is notable in the case of peptidases. The somewhat wide range of peptidases identified within our data set is mainly distributed among phages infecting bacteria with subtype A1 peptidoglycan. In phages that infect subtype A3 bacteria, the most common EAD is *CHAP*, which has been shown to function either as a NAM-amidase or as an endopeptidase and, in any case, seems to be specific for A3 peptidoglycan.

(iv) Fourth is the remarkably differential distribution of domain families among phages that infect either G+ or G−, together with the association of such domains with different physicochemical properties.

(v) The last point concerns the differential physicochemical properties between lysins from G+ and G−, which, conversely, allow prediction of the Gram group of the bacterial host of a given lysin based on its sequence. In this work, the trait of a positively charged patch at a C-terminal position was found to be widespread among at least a subpopulation of lysins from G−-infecting phages. This trait has been previously correlated with an improved ability to interact with the G− negatively charged OM. The higher values of other physicochemical variables in G− (aliphatic index and hydrophobic moment) also suggest an analogy of certain structural segments of G− lysins with AMPs.

These observations have clear implications for the design and development of lysin-based antimicrobials, from rational search (or design) of novel lysin parts to deriving AMPs from lysins sequences. A possible setup in which specific bacterial infections are tackled in an individualized manner based on a knowledge-driven, highly efficient synthetic biology platform for lysin-based antimicrobial production in the near future can be envisioned. The conclusions of this work can contribute to the consolidation of such a framework, together with the cutting-edge research currently being carried out in the field.

## MATERIALS AND METHODS

### Sequence database construction and curation.

Phage genomes were retrieved from the NCBI nucleotide database by searching complete phage genomes limited to several bacterial taxa of interest, mainly selected by clinical or epidemiological importance and availability. Those genomes were screened for gene products whose annotations could indicate that they are lytic enzymes. Therefore, keywords such as “lysin,” “lysozyme,” “murein,” “amidase,” “cell wall hydrolase,” “peptidase,” and “peptidoglycan” were used as inclusion criteria, while “structural,” “tail,” “holin,” “baseplate,” and “virion protein” were used as exclusion terms to avoid misidentifications. Associated information, such as the taxon of the bacterial host, amino acid sequence, annotations, phage denomination, and protein/genome unique identifiers, was also added to the database.

Curation included (i) a sequence length cutoff, established with a minimum of 50 and a maximum of 550 aa residues; (ii) a sequence identity cutoff using CD-HIT (70) with default parameters and a 98% identity cutoff value to avoid redundant entries; (iii) examination with PfamScan (expectation value cutoff = 10) (71, 72) to rule out sequences where no relevant significant hits were found (i.e., where no functional domains that would plausibly appear within phage lysins were detected); and (iv) bacterial host genus assignation to each entry based on literature and genome annotations. The Phobius server was used to add information on the presence of predicted N-terminal signal peptides (73). The complete lysin collection and PF hits are presented in Table S1 in the supplemental material and at Digital.CSIC (74).

### Physicochemical property prediction and analysis.

Prediction of physicochemical properties (net charge, aliphatic index, hydrophobicity, hydrophobic moment) based on the amino acid sequences retrieved were performed using the R package “Peptides” implementation (75). Dawson’s pK_a_ scale was used for prediction of net charge assuming a pH of 7.0 (76); the hydrophobicity scale was that proposed by Kyte and Doolittle (77), and hydrophobic moment was calculated as previously proposed (78) with a specified rotational angle of 100° (recommended angle for α-helix structures). An average value of the hydrophobic moment of each of the possible 11-aa helices within a given sequence is given whenever noted. Such properties were predicted in the whole sequences, in sequences quartiles (contiguous fragments of sequences that account in length each for a quarter of the whole sequence), or in peptides of 11 aa to provide either a global vision or more local information.

A random forest algorithm was used to check the ability of physicochemical properties to predict lysin sequences as from a G+- or G−-infecting phages. The R package “caret” was employed for creating, fitting, and testing the random forest, and further analyses on the model were performed using packages “pROC” and “randomForest.” The data set was randomly partitioned into a training subset (75% of all entries) and a testing subset. The training subset was used to fit the random forest parameters (namely, the randomly selected variables for each node, which was fixed at 4) by 5-fold cross-validation with 3 repeats. Then, the constructed random forest was validated using the previously defined testing subset. Alternatively, a second random forest model was constructed with additional variables (protein length, number of PF hits, and presence or absence of an N-terminal signal peptide), using the method described above.

### Sequence similarity networks.

SSNs were generated for visually assessing the similarity clustering of sequence sets. For this purpose, the Enzyme Similarity tool from the Enzyme Function Initiative server (EFI-EST) was employed (79). Briefly, this tool performs a local alignment from which every possible pair of sequences receives a score similar to the E value obtained from a typical BLAST analysis. A threshold score value was selected for each SSN so that below this threshold, sequence pairs were considered nonsimilar, and therefore, the pair would not be connected in the resulting representation. Scores were selected so that sequence pairs whose similarity was below 30 to 40% were deemed nonsimilar. The SSN graphs were produced using Cytoscape 3 with the yFiles organic layout (80).

### Statistical analysis.

Default methods for data representation implemented in the ggplot2 R package, such as kernel density estimation and GAM smoothing, were used throughout this work for data visualization (81). For comparison of nonnormal, heteroskedastic data populations, robust statistical methods were used (82). Specifically, a generalization of Welch’s test with trimmed means (default trimming level γ = 0.20) was used with a Bonferroni adjustment when multiple comparisons were performed. Effect sizes were estimated according to Wilcox and Tian’s ζ (83). A general interpretation for ζ is given in that reference, with values of around 0.10 indicating a small effect size, those around 0.30 a medium effect, and those of 0.50 and above a large one. A *P* value of ≤0.05 was considered significant. All robust methods used were from the implementation in the R package WRS2 (84).

### Data availability.

All data used in this work are available at the Digital.CSIC repository (http://hdl.handle.net/10261/221469) and in the supplemental material.
