# Supplementary material for: Sequence-Function Relationships in Phage-Encoded Bacterial Cell Wall Lytic Enzymes and Their Implications for Phage-Derived Product Design
Source: J Virol. 2021 Jun 24;95(14):e00321-21. doi: 10.1128/JVI.00321-21 (PMC8223927; doi:10.1128/JVI.00321-21)
Supplement: Supplemental file 2 — Table S2. Download JVI.00321-21-s0002.pdf, PDF file, 188 KB [file jvi.00321-21-s0002.pdf]

<u>Supplementary Information to:</u>

Sequence-function relationships in phage-encoded bacterial cell wall lytic enzymes and their implications for phage-derived products design

Roberto Vázquez[1,2*], Ernesto García[1,2], Pedro García[1,2]

*Correspondence: rvazquez@cib.csic.es

[1]Microbial and Plant Biotechnology Department, Centro de Investigaciones Biológicas Margarita Salas (CIB-CSIC), Madrid, Spain,

[2]Centro de Investigación Biomédica en Red de Enfermedades Respiratorias (CIBERES), Madrid, Spain

TABLE S2. PF families detected among our database and their proposed functions.

Name	Pfam	Interpro	Domain Type	Source/s	Activity	Notes
Amidase02_C	PF12123	IPR021976	CWBD	(1, 2)	Cell wall binding	Experimentally confirmed binding to *Bacillus* vegetative cells
Big_2	PF02368	IPR003343	CWBD	(3)	Cell wall binding	Similar to lectins; probable binding to carbohydrates.
CW_7	PF08230	IPR013168	CWBD	(4, 5)	Cell wall binding	Shown to bind the *N*-acetyl-D-glucosaminyl-(β1,4)-*N*-acetylmuramyl-L-alanyl-D-isoglutamine moiety of peptidoglycan
CW_binding_1	PF01473	IPR018337	CWBD	(6)	Cell wall binding	Known to bind choline residues at the teichoic acids of some mitis group streptococci
CW_binding_2	PF04122	IPR007253	CWBD	(7)	Cell wall binding	Clostridial cell surface motif; binds to surface glycans (specifically to highly conserved capsular polysaccharide PSII)
LGFP	PF08310	IPR013207	CWBD	(8)	Cell wall binding	Found in proteins from mycobacteria, next to esterase domains. Also found at C-terminal of glycoside hydrolase domains. Probable binding domain.
LysM	PF01476	IPR018392	CWBD	(9)	Cell wall binding	Recognizes polysaccharides containing N-acetylglucosamine residues, including peptidoglycan.
PG_binding_1	PF01471	IPR002477	CWBD	(10)	Cell wall binding	Appears at N- or C-terminal of enzymes involved in cell wall degradation. Presumed to bind peptidoglycan.
PG_binding_3	PF09374	IPR018537	CWBD	(11)	Cell wall binding	Potential peptidoglycan binding.
PSA_CBD	PF18341	IPR041341	CWBD	(12, 13)	Cell wall binding	Binding to cell wall teichoic acids. Determines specific binding to different *Listeria* serovars.
SH3_3	PF08239	IPR003646	CWBD		Cell wall binding	Putative binding domain.
SH3_5	PF08460	IPR003646	CWBD	(14-16)	Cell wall binding	Peptidoglycan binding (in staphylococcal phages, full specificity determined by the presence of pentaglycine bridge; in *Lactobacillus* it has been said to bind peptidoglycan stressing participation of glucosamine and low specificity)
SPOR	PF05036	IPR007730	CWBD	(17, 18)	Cell wall binding	Peptidoglycan binding domain found in proteins involved in sporulation and cell division. Displays preferent binding to septal peptidoglycan lacking stem peptides.
ZoocinA_TRD	PF16775	IPR031898	CWBD	(19)	Cell wall binding	Proposed to recognize A3α peptidoglycan, perhaps only with 2× alanine interpeptide bridges.
DUF3597	PF12200	IPR022016	CWBD	(13)	Cell wall binding	Probable binding domain. Similar to *Listeria* phage lysin Ply118 CWBD responsible for specific binding to cell wall elements. Found at C-terminal of hydrolytic domains.
3D	PF06725	IPR010611	EAD	(20)	Lytic Transglycosylase	
Amidase_2	PF01510	IPR002502	EAD	(21, 22)	NAM-amidase	
Amidase_3	PF01520	IPR002508	EAD	(23)	NAM-amidase	
Amidase_5	PF05382	IPR0002508	EAD	(24)	NAM-amidase	
CHAP	PF05257	IPR007921	EAD	(25-28)	Amidase or peptidase	It has been described to have either an amidase activity or an endopeptidase activity (cutting between D-Ala and the poly-Gly interpeptide bridge of staphylococci)
Cutinase	PF01083	IPR000675	EAD	(29, 30)	Esterase	Present in fungal enzymes that detach acetyl side groups from polysaccharides.
FSH1	PF03959	IPR005645	EAD	(31)	Esterase	
Glucosaminidase	PF01832	IPR002901	EAD	(32)	Glucosaminidase	
Glyco_hydro_108	PF05838	IPR008565	EAD	(33)	Muramidase	
Glyco_hydro_19	PF00182	IPR016283	EAD	(34)	Glucosaminidase (or muramidase)	Described as chitinase, probable glucosaminidase activity (or muramidase). Their only recognized function is as chitinases, which is hydrolysing bonds between *N*-acetyl-glucosamine residues. Since such homopolymer does not exist amongst bacteria, it is speculated that *Glyco_hydro_19* in phage lysins has a glycosidase, murolytic activity.
Glyco_hydro_25	PF01183	IPR002053	EAD	(35)	Muramidase	
GPW_gp25	PF04965	IPR007048	EAD	(36)	Muramidase	Virion associated lysin domain
Hydrolase_2	PF07486	IPR011105	EAD	(37)	Lytic Transglycosylase	
Muramidase	PF11860	IPR024408	EAD	(38)	Muramidase	
NLPC_P60	PF00877	IPR000064	EAD	(39, 40)	Peptidase	c-D-Glu-m-DAP endopeptidase
Peptidase_C39_2	PF13529	IPR039564	EAD	(41)	Peptidase	c-D-Glu-m-DAP endopeptidase
Peptidase_C93	PF06035	IPR010319	EAD	(42)	Peptidase	
Peptidase_M15_3	PF08291	IPR013230	EAD	(43)	Peptidase	
Peptidase_M15_4	PF13539	IPR039561	EAD	(41, 44)	Peptidase	D-Ala-m-DAP/m-DAP-m-DAP / L-Ala-D-Glu endopeptidase
Peptidase_M23	PF01551	IPR016047	EAD	(45-47)	Peptidase	Peptidase (Gly-Gly interpeptide bridge; Ala-Glu bonds)
Pesticin	PF16754	IPR031922	EAD	(48)	Muramidase	
Phage_lysozyme	PF00959	IPR002196	EAD	(49)	Muramidase	
Phage_lysozyme2	PF18013	IPR041219	EAD	(50)	Muramidase	Virion associated lysin domain
Prok-JAB	PF14464	IPR028090	EAD	(51)	Peptidase	

SLT	PF01464	IPR008258	EAD	(52)	Lytic Transglycosylase	
Transglycosylase	PF06737	IPR010618	EAD		Lytic Transglycosylase	
PE-PPE	PF08237	IPR013228	EAD	(53)	Esterase	C-terminal to mycobacterial PE and PPE proteins, possible esterase based on active site homology
Prophage_tail	PF06605	IPR010572	EAD		Peptidase	Virion associated endopeptidase domain
Gp5_C	PF06715	IPR010609	Structural		Structural	
Gp5_OB	PF06714	IPR009590	Structural		Structural	

REFERENCES

1. Fujinami Y, Hirai Y, Sakai I, Yoshino M, Yasuda J. 2007. Sensitive detection of *Bacillus anthracis* using a binding protein originating from γ-phage. Microbiol Immunol 51:163–169.
2. Kikkawa H, Fujinami Y, Suzuki S, Yasuda J. 2007. Identification of the amino acid residues critical for specific binding of the bacteriolytic enzyme of γ-phage, PlyG, to *Bacillus anthracis*. Biochem Biophys Res Commun 363:531–535.
3. Kelly G, Prasannan S, Daniell S, Fleming K, Frankel G, Dougan G, Connerton I, Matthews S. 1999. Structure of the cell-adhesion fragment of intimin from enteropathogenic *Escherichia coli*. Nat Struct Biol 6:313–318.
4. Bustamante N, Campillo NE, García E, Gallego C, Pera B, Diakun GP, Sáiz JL, García P, Díaz JF, Menéndez M. 2010. Cpl-7, a lysozyme encoded by a pneumococcal bacteriophage with a novel cell wall-binding motif. J Biol Chem 285:33184–33196.
5. Bustamante N, Iglesias-Bexiga M, Bernardo-García N, Silva-Martín N, García G, Campanero-Rhodes MA, García E, Usón I, Buey RM, García P, Hermoso JA, Bruix M, Menéndez M. 2017. Deciphering how Cpl-7 cell wall-binding repeats recognize the bacterial peptidoglycan. Sci Rep 7:16494.
6. Hermoso JA, Monterroso B, Albert A, Galán B, Ahrazem O, García P, Martínez-Ripoll M, García JL, Menéndez M. 2003. Structural basis for selective recognition of pneumococcal cell wall by modular endolysin from phage Cp-1. Structure 11:1239–1249.
7. Willing SE, Candela T, Shaw HA, Seager Z, Mesnage S, Fagan RP, Fairweather NF. 2015. *Clostridium difficile* surface proteins are anchored to the cell wall using CWB2 motifs that recognise the anionic polymer PSII. Mol Microbiol 96:596–608.
8. Brand S, Niehaus K, Pühler A, Kalinowski J. 2003. Identification and functional analysis of six mycolyltransferase genes of *Corynebacterium glutamicum* ATCC 13032: the genes *cop1, cmt1,* and *cmt2* can replace each other in the synthesis of trehalose dicorynomycolate, a component of the mycolic acid layer of the cell envelope. Arch Microbiol 180:33–44.
9. Mesnage S, Dellarole M, Baxter NJ, Rouget JB, Dimitrov JD, Wang N, Fujimoto Y, Hounslow AM, Lacroix-Desmazes S, Fukase K, Foster SJ, Williamson MP. 2014. Molecular basis for bacterial peptidoglycan recognition by LysM domains. Nat Commun 5:4269.
10. Maestro B, Sanz JM. 2016. Choline binding proteins from *Streptococcus pneumoniae*: a dual role as enzybiotics and targets for the design of new antimicrobials. Antibiotics (Basel) 5:21.
11. Pei J, Grishin NV. 2005. COG3926 and COG5526: a tale of two new lysozyme-like protein families. Protein Sci 14:2574–2581.
12. Korndörfer IP, Danzer J, Schmelcher M, Zimmer M, Skerra A, Loessner MJ. 2006. The crystal structure of the bacteriophage PSA endolysin reveals a unique fold responsible for specific recognition of *Listeria* cell walls. J Mol Biol 364:678–689.
13. Loessner MJ, Kramer K, Ebel F, Scherer S. 2002. C-terminal domains of *Listeria monocytogenes* bacteriophage murein hydrolases determine specific recognition and high-affinity binding to bacterial cell wall carbohydrates. Mol Microbiol 44:335–349.
14. Lu JZ, Fujiwara T, Komatsuzawa H, Sugai M, Sakon J. 2006. Cell wall-targeting domain of glycylglycine endopeptidase distinguishes among peptidoglycan cross-bridges. J Biol Chem 281:549–558.
15. Gründling A, Schneewind O. 2006. Cross-linked peptidoglycan mediates lysostaphin binding to the cell wall envelope of *Staphylococcus aureus*. J Bacteriol 188:2463–2472.
16. Beaussart A, Rolain T, Duchêne M-C, El-Kirat-Chatel S, Andre G, Hols P, Dufrêne YF. 2013. Binding mechanism of the peptidoglycan hydrolase Acm2: low affinity, broad specificity. Biophys J 105:620–629.
17. Yahashiri A, Jorgenson MA, Weiss DS. 2015. Bacterial SPOR domains are recruited to septal peptidoglycan by binding to glycan strands that lack stem peptides. Proc Natl Acad Sci U S A 112:11347–11352.
18. Alcorlo M, Dik DA, De Benedetti S, Mahasenan KV, Lee M, Domínguez-Gil T, Hesek D, Lastochkin E, López D, Boggess B, Mobashery S, Hermoso JA. 2019. Structural basis of denuded glycan recognition by SPOR domains in bacterial cell division. Nat Commun 10:5567.
19. Chen Y, Simmonds RS, Timkovich R. 2013. Proposed docking interface between peptidoglycan and the target recognition domain of zoocin A. Biochem Biophys Res Commun 441:297–300.

20. van Straaten KE, Dijkstra BW, Vollmer W, Thunnissen A-MWH. 2005. Crystal structure of MltA from *Escherichia coli* reveals a unique lytic transglycosylase fold. J Mol Biol 352:1068–1080.
21. Lee M, Artola-Recolons C, Carrasco-López C, Martínez-Caballero S, Hesek D, Spink E, Lastochkin E, Zhang W, Hellman LM, Boggess B, Hermoso JA, Mobashery S. 2013. Cell-wall remodeling by the zinc-protease AmpDh3 from *Pseudomonas aeruginosa*. J Am Chem Soc 135:12604–12607.
22. Martínez-Caballero S, Lee M, Artola-Recolons C, Carrasco-López C, Hesek D, Spink E, Lastochkin E, Zhang W, Hellman LM, Boggess B, Mobashery S, Hermoso JA. 2013. Reaction products and the X-ray structure of AmpDh2, a virulence determinant of *Pseudomonas aeruginosa*. J Am Chem Soc 135:10318–10321.
23. Büttner FM, Zoll S, Nega M, Götz F, Stehle T. 2014. Structure-function analysis of *Staphylococcus aureus* amidase reveals the determinants of peptidoglycan recognition and cleavage. J Biol Chem 289:11083–11094.
24. Garcia P, Garcia E, Ronda C, Lopez R, Tomasz A. 1983. A phage-associated murein hydrolase in *Streptococcus pneumoniae* infected with bacteriophage Dp-1. J Gen Microbiol 129:489–497.
25. Bateman A, Rawlings ND. 2003. The CHAP domain: a large family of amidases including GSP amidase and peptidoglycan hydrolases. Trends Biochem Sci 28:234–237.
26. Rigden DJ, Jedrzejas MJ, Galperin MY. 2003. Amidase domains from bacterial and phage autolysins define a family of γ-D,L-glutamate-specific amidohydrolases. Trends Biochem Sci 28:230–234.
27. Gu J, Feng Y, Feng X, Sun C, Lei L, Ding W, Niu F, Jiao L, Yang M, Li Y, Liu X, Song J, Cui Z, Han D, Du C, Yang Y, Ouyang S, Liu Z-J, Han W. 2014. Structural and biochemical characterization reveals LysGH15 as an unprecedented "EF-hand-like" calcium-binding phage lysin. PLoS Pathog 10:e1004109.
28. Rossi P, Aramini JM, Xiao R, Chen CX, Nwosu C, Owens LA, Maglaqui M, Nair R, Fischer M, Acton TB, Honig B, Rost B, Montelione GT. 2009. Structural elucidation of the Cys-His-Glu-Asn proteolytic relay in the secreted CHAP domain enzyme from the human pathogen *Staphylococcus saprophyticus*. Proteins 74:515–519.
29. Hakulinen N, Tenkanen M, Rouvinen J. 2000. Three-dimensional structure of the catalytic core of acetylxylan esterase from *Trichoderma reesei*: insights into the deacetylation mechanism. J Struct Biol 132:180–190.
30. Martinez C, De Geus P, Lauwereys M, Matthyssens G, Cambillau C. 1992. *Fusarium solani* cutinase is a lipolytic enzyme with a catalytic serine accessible to solvent. Nature 356:615–618.
31. Xu W, Chooi Y-H, Choi JW, Li S, Vederas JC, Da Silva NA, Tang Y. 2013. LovG: the thioesterase required for dihydromonacolin L release and lovastatin nonaketide synthase turnover in lovastatin biosynthesis. Angew Chem Int Ed English 52:6472–6475.
32. Inagaki N, Iguchi A, Yokoyama T, Yokoi K-j, Ono Y, Yamakawa A, Taketo A, Kodaira K-I. 2009. Molecular properties of the glucosaminidase AcmA from *Lactococcus lactis* MG1363: Mutational and biochemical analyses. Gene 447:61–71.
33. Stojković EA, Rothman-Denes LB. 2007. Coliphage N4 *N*-acetylmuramidase defines a new family of murein hydrolases. J Mol Biol 366:406–419.
34. Oliveira H, Melo LDR, Santos SB, Nóbrega FL, Ferreira EC, Cerca N, Azeredo J, Kluskens LD. 2013. Molecular aspects and comparative genomics of bacteriophage endolysins. J Virol 87:4558–4570.
35. Davies G, Henrissat B. 1995. Structures and mechanisms of glycosyl hydrolases. Structure 3:853–859.
36. Szewczyk B, Bienkowska-Szewczyk K, Kozloff LM. 1986. Identification of T4 gene 25 product, a component of the tail baseplate, as a 15K lysozyme. Mol Gen Genet 202:363–367.
37. Li Y, Jin K, Setlow B, Setlow P, Hao B. 2012. Crystal structure of the catalytic domain of the *Bacillus cereus* SleB protein, important in cortex peptidoglycan degradation during spore germination. J Bacteriol 194:4537–4545.
38. Rodríguez-Rubio L, Gerstmans H, Thorpe S, Mesnage S, Lavigne R, Briers Y. 2016. DUF3380 domain from a *Salmonella* phage endolysin shows potent *N*-acetylmuramidase activity. Appl Environ Microbiol 82:4975–4981.
39. Anantharaman V, Aravind L. 2003. Evolutionary history, structural features and biochemical diversity of the NlpC/P60 superfamily of enzymes. Genome Biol 4:R11.
40. Xu Q, Abdubek P, Astakhova T, Axelrod HL, Bakolitsa C, Cai X, Carlton D, Chen C, Chiu HJ, Chiu M, Clayton T, Das D, Deller MC, Duan L, Ellrott K, Farr CL, Feuerhelm J, Grant JC, Grzechnik A, Han GW, Jaroszewski L, Jin KK, Klock HE, Knuth MW, Kozbial

P, Krishna SS, Kumar A, Lam WW, Marciano D, Miller MD, Morse AT, Nigoghossian E, Nopakun A, Okach L, Puckett C, Reyes R, Tien HJ, Trame CB, van den Bedem H, Weekes D, Wooten T, Yeh A, Hodgson KO, Wooley J, Elsliger MA, Deacon AM, Godzik A, Lesley SA, Wilson IA. 2010. Structure of the γ-D-glutamyl-L-diamino acid endopeptidase YkfC from *Bacillus cereus* in complex with L-Ala-γ-D-Glu: insights into substrate recognition by NlpC/P60 cysteine peptidases. Acta Crystallogr Sect F Struct Biol Cryst Commun 66:1354–1364.

41. Payne KM, Hatfull GF. 2012. Mycobacteriophage endolysins: diverse and modular enzymes with multiple catalytic activities. PLoS One 7:e34052.
42. Ginalski K, Kinch L, Rychlewski L, Grishin NV. 2004. BTLCP proteins: a novel family of bacterial transglutaminase-like cysteine proteinases. Trends Biochem Sci 29:392–395.
43. Bochtler M, Odintsov SG, Marcyjaniak M, Sabala I. 2004. Similar active sites in lysostaphins and D-Ala-D-Ala metallopeptidases. Protein Sci 13:854–861.
44. Fukushima T, Yao Y, Kitajima T, Yamamoto H, Sekiguchi J. 2007. Characterization of new L,D-endopeptidase gene product CwlK (previous YcdD) that hydrolyzes peptidoglycan in *Bacillus subtilis*. Mol Genet Genomics 278:371–383.
45. Schneewind O, Fowler A, Faull KF. 1995. Structure of the cell wall anchor of surface proteins in *Staphylococcus aureus*. Science 268:103–106.
46. Grabowska M, Jagielska E, Czapinska H, Bochtler M, Sabala I. 2015. High resolution structure of an M23 peptidase with a substrate analogue. Sci Rep 5:14833.
47. Horsburgh GJ, Atrih A, Foster SJ. 2003. Characterization of LytH, a differentiation-associated peptidoglycan hydrolase of *Bacillus subtilis* involved in endospore cortex maturation. J Bacteriol 185:3813–3820.
48. Patzer SI, Albrecht R, Braun V, Zeth K. 2012. Structural and mechanistic studies of pesticin, a bacterial homolog of phage lysozymes. J Biol Chem 287:23381–23396.
49. Mooers BHM, Matthews BW. 2006. Extension to 2268 atoms of direct methods in the *ab initio* determination of the unknown structure of bacteriophage P22 lysozyme. Acta Crystallogr D Biol Crystallogr 62:165–176.
50. Xiang Y, Morais MC, Cohen DN, Bowman VD, Anderson DL, Rossmann MG. 2008. Crystal and cryoEM structural studies of a cell wall degrading enzyme in the bacteriophage φ29 tail. Proc Natl Acad Sci U S A 105:9552–9557.
51. Iyer LM, Burroughs AM, Aravind L. 2006. The prokaryotic antecedents of the ubiquitin-signaling system and the early evolution of ubiquitin-like β-grasp domains. Genome Biol 7:R60.
52. Williams AH, Wheeler R, Thiriau C, Haouz A, Taha M-K, Boneca IG. 2017. Bulgecin A: the key to a broad-spectrum inhibitor that targets lytic transglycosylases. Antibiotics (Basel) 6:8.
53. Sultana R, Tanneeru K, Guruprasad L. 2011. The PE-PPE domain in mycobacterium reveals a serine α/β hydrolase fold and function: an *in-silico* analysis. PLoS One 6:e16745.
